# X-ray crystal structure of [*L*_2_Ag_3_]^+^[OTf]^−^·5C_6_D_6_: a monoanionic bis­phosphinimine ligand supported tris­ilver complex

**DOI:** 10.1107/S2056989021009221

**Published:** 2021-09-17

**Authors:** Sam L. Drescher, Christopher P. Forfar, René T. Boeré, Paul G. Hayes

**Affiliations:** a Department of Chemistry and Biochemistry, University of Lethbridge, 4401, University Drive West, Lethbridge, AB, Canada, T1K 3M4

**Keywords:** X-ray crystal structure, phosphinimine, tris­ilver, monoanionic

## Abstract

The compound bis­{μ_3_-2,5-bis­[*N*-(4-iso­propyl­phen­yl)-*P*,*P*-di­phenyl­phospho­r­im­i­do­yl]pyrrol-1-ido-κ^3^ *N*:*N*′:*N*′′}tris­ilver(I) tri­fluoro­methane­sulfonate deuterated benzene penta­solvate, [Ag_3_(C_46_H_44_N_3_P_2_)_2_](CF_3_O_3_S)·5C_6_D_6_, (I), was synthesized from two equivalents of Na*L* [*L* = 2,5-(4-^*i*^PrC_6_H_4_N=PPh_2_)C_4_H_2_N] and three equivalents of AgOTf (OTf = OSO_2_CF_3_). High-quality crystals of (I) formed with five deuterated benzene solvent mol­ecules in the asymmetric unit. Notably, the bond lengths and angles between the three silver atoms are markedly different, unlike in most other reported tris­ilver complexes that tend to form three equivalent metal centers. Additionally, to the best of our knowledge, this is the first report of a tris­ilver complex with six nitro­gen donors from two bis­phosphinimine ligands.

## Abstract 2

A tris­ilver complex stabilized by two monoanionic bis­phosphinimine ligands is reported. Noteworthy asymmetry at the cluster core is observed. This is the first example of a tris­ilver complex supported by monoanionic bis­phosphinimine ligands.

## Chemical context   

Silver clusters have been reported extensively in the literature, and tris­ilver clusters tend to be particularly common. Tang and Zhao recently demonstrated that a tris­ilver cluster was highly favoured with six nitro­gen donors, even when ten equivalents of silver triflate were used (Tang & Zhao, 2020 ▸). Although there have been a few examples of silver complexes supported by phosphinimine ligands [Cambridge Structural Database, Version 5.41, update of December 2020; Groom *et al.*, 2016 ▸; CSD refcodes OHILEZ and OHILUP (Aguirre Quintana *et al.*, 2020 ▸), LAHCII (Brown *et al.*, 2010 ▸), UKEGUO (Thirumoorthi *et al.*, 2016 ▸) AVAPEV and AVAPIZ (Jha *et al.*, 2021 ▸)], we are not aware of any tris­ilver complexes with bis­phosphinimine ligands, as is the case with the structure reported herein. We have, however, reported numerous studies that employ a variety of pyrrole-based bis­phosphinimine ligands with a broad array of metals (*e.g*. Sm, Rh, Th) across the periodic table [BUJWAH (Dickie *et al.*, 2020 ▸), YUXSOB, YUXSUH and YUXTAO (Hänninen *et al.*, 2016 ▸), LOTYIF (Knott *et al.*, 2017 ▸), GIRRIL, GIRROR and GIRSEI (MacNeil *et al.* 2018 ▸), ROGFUQ, ROGGAX and ROGGEB (Zamora *et al.* 2014 ▸)].

## Structural commentary   

**Figure 1:**
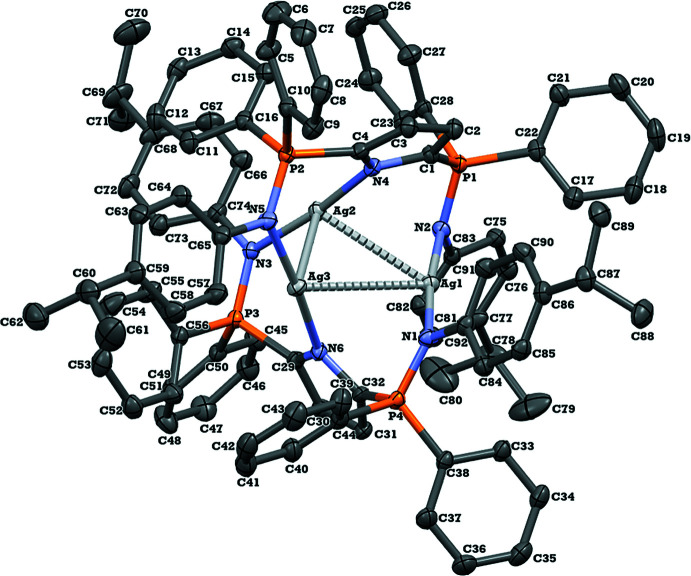
Displacement ellipsoid plot (50% probability) of (I) showing the atomic labelling scheme. Hydrogen atoms, the trifluoromethanesulfonate counter-ion, and the deuterated benzene solvent mol­ecules have been removed for clarity.

The title compound [*L*_2_Ag_3_]^+^[OTf]^−^·5C_6_D_6_ (*L* = 2,5-(4-^*i*^PrC_6_H_4_N=PPh_2_)C_4_H_2_N; OTf = OSO_2_CF_3_) (I) has three silver cations coordinated by two tridentate, anionic, pyrrole-based bis­phoshinimine ligands (Fig. 1 ▸). The tris­ilver complex is highly asymmetric with all three silver distances significantly different (Table 1 ▸). The likely cause of this is that two of the silver ions are coordinated to one anionic pyrrole nitro­gen (N4 and N6) and one phosphinimine nitro­gen (N3 and N5), whereas the remaining silver atom is coordinated to two phosphinimine nitro­gens (N1 and N2) (Fig. 1 ▸). The Ag—N bond lengths are similar and range from 2.127 (2) to 2.173 (2) Å, significantly shorter than the average found in a CSD search of 2.318 (20) Å. As a result of the silver atoms being coordinated to different nitro­gen atoms, the Ag1—Ag3 and Ag1—Ag2 distances are substanti­ally longer than Ag2—Ag3, which in turn causes an acute Ag2—Ag1—Ag3 angle of 52.186 (5), far less than the average value of 60.0 (2)° found in the CSD.
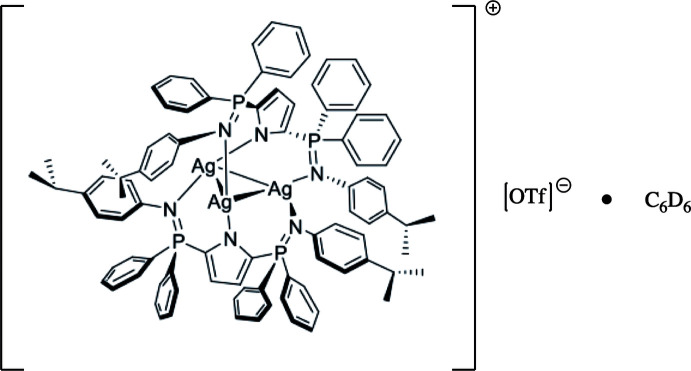


There are numerous examples of tris­ilver complexes with six nitro­gen donors ranging from *N*-heterocyclic carbenes to cryptates [CSD refcodes ALEZEW (Catalano & Malwitz, 2003 ▸), ABISEK (Catalano *et al.*, 2004 ▸), and ACUWAW (McKee *et al.*, 2001 ▸)]. Ag—Ag bond distances in tris­ilver complexes with six nitro­gen donors average around 2.95 (2) Å (CSD), shorter than the distances between Ag1—Ag2 [3.2177 (2) Å] and Ag1—Ag3 [3.3165 (2) Å], but longer than the shorter distance of Ag2—Ag3 [2.8753 (3) Å]. Throughout the aforementioned examples we are not aware of any complexes that have such a significant difference in bond lengths when comparing all three silver distances. For example, the shortest [2.8487 (11) Å] and longest [3.0896 (11) Å] bond lengths in another tris­ilver complex reported by McKee *et al.* (2001 ▸) differ by 0.24 Å, compared to a difference of 0.44 Å in the present complex (Table 1 ▸).

Compared to the neutral, protonated, ligand precursor H*L*, key bond distances and angles are similar with some slight distortions. The C—N—C bond angle of the pyrrole ring changes from 109.0 (2)° (CSD refcode NAYMIL; Johnson *et al.* 2012 ▸) to 105.6 (2)°, likely as a consequence of coordination to Ag2 and Ag3 (Table 1 ▸). Additionally, although the C—P—N bond angles remain significantly different on one side of the ligand to the other, the C—P—N angle of the phosphinimine functionalities coordinated to Ag2 and Ag3 are significantly smaller than that of H*L* [111.63 (3)° from 119.37 (12)°]. There is not a substantial difference between the more acute C—P—N bond angle in H*L* [106.29 (12)°] compared to the C—P—N angle of the phosphinimine moieties coordinated to Ag1 [105.3 (6)°]. Lastly, there is no major change in the P=N distance of the phosphinimine groups [1.61 (3) Å from 1.57 (7) Å].

Johnson *et al.* (2009 ▸) reported a tris­ilver complex Ag_3_ *L*_3_ {*L* = μ_2_-1,3-bis­[2,6-(diiso­propyl­phen­yl)triazenide]} and described their complex as a silver triangle possessing equilateral geometry such that their Ag—Ag bond distances are approximately the same within error (CSD refcode OGOHIC). The current compound does not show this equilateral geometry. McKee *et al.* (2001 ▸) describe their tris­ilver complex (CSD refcode ACUWAW) as ‘near-linear’ without considering the Ag⋯Ag short contacts. The current complex (I) also shows a distorted linear geometry when only considering Ag—N contacts with N—Ag—N angles averaging 172.8 (2)° (Table 1 ▸).

## Supra­molecular features   

**Figure 2:**
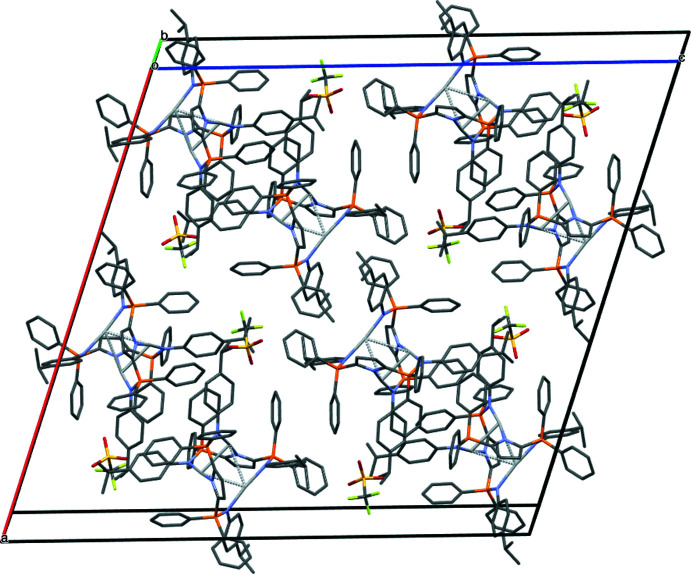
Packing diagram of (I) viewed down the *b* axis with deuterated benzene solvent mol­ecules removed for clarity.

**Figure 3:**
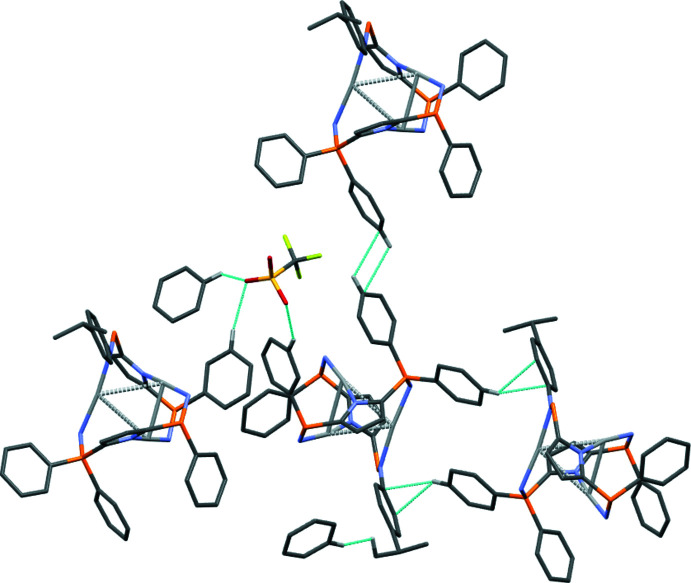
Representation of short contacts with only key hydrogen atoms and deuterated benzene solvent mol­ecules shown for clarity

The title compound recrystallized in the monoclinic space group *C*2/*c* with five deuterated benzene solvent mol­ecules in the asymmetric unit (Fig. 2 ▸). Other researchers have found their silver complexes to crystallize as a benzene solvate [CSD refcodes HAJQER (Cook *et al.*, 2016 ▸) and AFOJOX (Li *et al.* 2018 ▸)]; additionally, toluene solvent was present in crystals of a rhodium complex stabilized by an analogous pyrrole ligand (CSD refcode GIRSEI; MacNeil *et al.* 2018 ▸). Furthermore, Cook *et al.* found that the solvent mol­ecules in the lattice of their hexa­silver complex had inter­actions with the phenyl groups in the ligand. The only short contact with the solvent mol­ecules in (I) is an inter­action with one hydrogen on one *para*-iso­propyl­phenyl (Pipp) group (Table 2 ▸, Fig. 3 ▸). Other short contacts are between phenyl groups on phospho­rus and the Pipp groups (Table 2 ▸, Fig. 3 ▸).

Numerous reports of tris­ilver complexes contain inter­actions between the silver atoms and the triflate counter-ion, although they are often weak inter­actions [CSD refcodes ACUWAW (McKee *et al.*, 2001 ▸), MEMSOM (Su *et al.*, 2000 ▸) and VIGNEF (Martin *et al.*, 2007 ▸)]. No silver–anion inter­actions were observed for (I). Consideration of a space-filling model of the asymmetric unit reveals that the bulky monoanionic pincer-ligand shields the silver atoms from any inter­actions with the triflate oxygen atoms. These oxygen atoms do, however, display short contacts with *meta* hydrogens on two separate phenyl groups (H14 and H52) as well as one hydrogen on a deuterated benzene solvent mol­ecule (H*M*), as shown in Fig. 3 ▸.

## Synthesis and crystallization   

In an NMR tube, two equivalents of Na*L* [*L* = 2,5-(4-^*i*^PrC_6_H_4_N=PPh_2_)C_4_H_2_N] and three equivalents of AgOTf were dissolved in benzene-*d*_6_. Crystals were grown in the NMR tube from benzene-*d*_6_. The synthesis of Na*L* has been previously published and utilizes a modified Staudinger reaction (Hänninen *et al.*, 2016 ▸; Staudinger & Meyer, 1919 ▸).

## Refinement   

Crystal data, data collection and structure refinement details are summarized in Table 3 ▸. The structure was solved with intrinsic phasing using *SHELXT* and refined with *SHELXL*. Problems with large residual peaks indicated the need to investigate the reciprocal lattice. Evidence of a cracked crystal with a four-component multi-crystal model was developed in *CrysAlis PRO* v41.113a. Twin refinement and finalization produced an HKLF4 file with completeness greater than 99% and acceptable *I*/σ. A sufficient refinement was obtained and removed the large residual peaks. After the silver cluster and tri­fluoro­methane­sulfonate were accurately modelled, no less than five deuterated benzene solvent mol­ecules were revealed in difference maps, two of which were well ordered and three were positionally disordered. The latter deuterated benzene solvent mol­ecules were split into two independent units and SADI and RIGU restraints were applied during refinement. Additionally, one of the solvent mol­ecules was restrained with an ISOR restraint.

## Supplementary Material

Crystal structure: contains datablock(s) I. DOI: 10.1107/S2056989021009221/zv2008sup1.cif

Structure factors: contains datablock(s) I. DOI: 10.1107/S2056989021009221/zv2008Isup7.hkl

CCDC reference: 2108219

Additional supporting information:  crystallographic information; 3D view; checkCIF report

## Figures and Tables

**Table 1:** Selected geometric parameters (Å, °)

Ag1—Ag2	3.2177 (2)	P1—N2	1.610 (2)
Ag2—Ag3	2.8753 (3)	P2—N5	1.610 (2)
Ag1—Ag3	3.3165 (2)	P3—N3	1.608 (2)
Ag1—N1	2.168 (2)	P4—N1	1.610 (2)
Ag1—N2	2.173 (2)	N1—C92	1.413 (3)
Ag2—N3	2.127 (2)	N2—C83	1.416 (3)
Ag2—N4	2.129 (2)	N3—C74	1.425 (3)
Ag3—N5	2.136 (2)	N5—C65	1.421 (3)
Ag3—N6	2.130 (2)		
			
Ag1—Ag2—Ag3	65.677 (6)	N1—Ag1—Ag2	121.87 (6)
Ag2—Ag1—Ag3	52.186 (5)	N1—Ag1—Ag3	69.92 (6)
Ag2—Ag3—Ag1	62.138 (6)	N2—Ag1—Ag2	64.04 (6)
N1—Ag1—N2	173.61 (8)	N2—Ag1—Ag3	116.14 (6)
N3—Ag2—N4	170.50 (8)	N3—Ag2—Ag1	120.57 (6)
N5—Ag3—N6	174.27 (8)	N3—Ag2—Ag3	92.63 (6)
C1—N4—C4	105.6 (2)	N5—Ag3—Ag1	121.38 (6)
C29—N6—C32	105.3 (2)	N5—Ag3—Ag2	95.83 (6)
C1—P1—N2	104.88 (12)	N4—Ag2—Ag1	65.22 (6)
C4—P2—N5	111.60 (11)	N4—Ag2—Ag3	83.00 (6)
C29—P3—N3	111.65 (11)	N6—Ag3—Ag1	61.87 (5)
C32—P4—N1	105.67 (11)	N6—Ag3—Ag2	81.53 (6)

**Table 2:** Summary of short contacts (Å) for (I)

Atoms	Length	Symmetry operation
H35⋯C75	2.728	1 − *x*, 1 − *y*, 1 − *z*
H35⋯C83	2.581	1 − *x*, 1 − *y*, 1 − *z*
H52⋯O3	2.548	*x*, *y*, *z*
H14⋯O1	2.601	{3\over 2} − *x*, −{1\over 2} + *y*, {3\over 2} − *z*
H80*A*⋯H*Q*	2.299	*x*, 1 + *y*, *z*
O1⋯H*M*	2.585	{3\over 2} − *x*, {1\over 2} + *y*, {3\over 2} − *z*

**Table 3:** Experimental details

Crystal data	
Chemical formula	[Ag_3_(C_46_H_44_N_3_P_2_)_2_](CF_3_O_3_S)·5C_6_D_6_
*M*_r_	2264.78
Crystal system, space group	Monoclinic, *C*2/*c*
Temperature (K)	100
*a*, *b*, *c* (Å)	35.6053 (2), 16.8381 (1), 37.5880 (3)
β (°)	108.548 (1)
*V* (Å^3^)	21364.4 (3)
*Z*	8
Radiation type	Cu *K*α
μ (mm^−1^)	5.60
Crystal size (mm)	0.17 × 0.13 × 0.07

Data collection	
Diffractometer	Rigaku SuperNova, Dual, Cu at home/near, Pilatus 200K
Absorption correction	Multi-scan (*CrysAlis PRO*; Rigaku OD, 2021 ▸)
*T*_min_, *T*_max_	0.386, 1.000
No. of measured, independent and observed [*I* > 2σ(*I*)] reflections	115297, 22743, 20031
*R*_int_	0.054
(sin θ/λ)_max_ (Å^−1^)	0.638

Refinement	
*R*[*F*^2^ > 2σ(*F* ^2^)], *wR*(*F* ^2^), *S*	0.038, 0.098, 1.03
No. of reflections	22743
No. of parameters	1461
No. of restraints	336
H-atom treatment	H-atom parameters constrained
Δρ_max_, Δρ_min_ (e Å^−3^)	1.11, −0.83

Computer programs: *CrysAlis PRO* (Rigaku OD, 2021 ▸), *SHELXT2018/2* (Sheldrick, 2015*a* ▸), *SHELXL2018/3* (Sheldrick, 2015*b* ▸), *Mercury* (Macrae *et al.*, 2020 ▸) and *OLEX2* (Dolomanov *et al.*, 2009 ▸).

## supplementary crystallographic information

### Crystal data

**Table:**

[Ag_3_(C_46_H_44_N_3_P_2_)_2_](CF_3_O_3_S)·5C_6_D_6_	*F*(000) = 9328
*M**_r_* = 2264.78	*D*_x_ = 1.408 Mg m^−^^3^
Monoclinic, *C*2/*c*	Cu *K*α radiation, λ = 1.54184 Å
*a* = 35.6053 (2) Å	Cell parameters from 64197 reflections
*b* = 16.8381 (1) Å	θ = 2.6–79.8°
*c* = 37.5880 (3) Å	µ = 5.60 mm^−^^1^
β = 108.548 (1)°	*T* = 100 K
*V* = 21364.4 (3) Å^3^	Prism, clear colourless
*Z* = 8	0.17 × 0.13 × 0.07 mm

### Data collection

**Table:**

Rigaku SuperNova, Dual, Cu at home/near, Pilatus 200K diffractometer	22743 independent reflections
Radiation source: micro-focus sealed X-ray tube, SuperNova (Cu) X-ray Source	20031 reflections with *I* > 2σ(*I*)
Mirror monochromator	*R*_int_ = 0.054
Detector resolution: 5.8140 pixels mm^-1^	θ_max_ = 79.8°, θ_min_ = 2.5°
ω scans	*h* = −45→45
Absorption correction: multi-scan (CrysAlisPro; Rigaku OD, 2021)	*k* = −16→21
*T*_min_ = 0.386, *T*_max_ = 1.000	*l* = −47→46
115297 measured reflections	

### Refinement

**Table:**

Refinement on *F*^2^	336 restraints
Least-squares matrix: full	Hydrogen site location: inferred from neighbouring sites
*R*[*F*^2^ > 2σ(*F*^2^)] = 0.038	H-atom parameters constrained
*wR*(*F*^2^) = 0.098	*w* = 1/[σ^2^(*F*_o_^2^) + (0.0481*P*)^2^ + 67.3826*P*] where *P* = (*F*_o_^2^ + 2*F*_c_^2^)/3
*S* = 1.03	(Δ/σ)_max_ = 0.005
22743 reflections	Δρ_max_ = 1.11 e Å^−^^3^
1461 parameters	Δρ_min_ = −0.83 e Å^−^^3^

### Special details

**Table:**

Geometry. All esds (except the esd in the dihedral angle between two l.s. planes) are estimated using the full covariance matrix. The cell esds are taken into account individually in the estimation of esds in distances, angles and torsion angles; correlations between esds in cell parameters are only used when they are defined by crystal symmetry. An approximate (isotropic) treatment of cell esds is used for estimating esds involving l.s. planes.

### Fractional atomic coordinates and isotropic or equivalent isotropic displacement parameters (Å^2^)

**Table:**

	*x*	*y*	*z*	*U*_iso_*/*U*_eq_	Occ. (<1)
Ag1	0.59842 (2)	0.31842 (2)	0.56933 (2)	0.02035 (5)	
Ag2	0.68778 (2)	0.30268 (2)	0.62522 (2)	0.01922 (5)	
Ag3	0.62866 (2)	0.31135 (2)	0.66234 (2)	0.01962 (5)	
P1	0.65635 (2)	0.22941 (4)	0.53491 (2)	0.01814 (12)	
P2	0.66732 (2)	0.13673 (4)	0.67524 (2)	0.01868 (12)	
P3	0.69577 (2)	0.47567 (4)	0.66692 (2)	0.01733 (12)	
P4	0.54038 (2)	0.39677 (4)	0.60798 (2)	0.01829 (12)	
N1	0.54781 (6)	0.31437 (13)	0.58956 (6)	0.0208 (4)	
N2	0.64465 (6)	0.31839 (13)	0.54303 (6)	0.0195 (4)	
N3	0.71843 (6)	0.40379 (13)	0.65425 (6)	0.0192 (4)	
N4	0.65940 (6)	0.19274 (12)	0.60512 (6)	0.0191 (4)	
N5	0.64398 (7)	0.20142 (13)	0.69177 (6)	0.0207 (4)	
N6	0.61919 (6)	0.42496 (12)	0.63605 (5)	0.0178 (4)	
C1	0.64410 (7)	0.16980 (15)	0.56831 (7)	0.0194 (5)	
C2	0.62540 (8)	0.09629 (16)	0.56516 (7)	0.0222 (5)	
H2	0.613015	0.067591	0.542723	0.027*	
C3	0.62856 (8)	0.07323 (16)	0.60191 (7)	0.0225 (5)	
H3	0.618567	0.025902	0.609321	0.027*	
C4	0.64938 (7)	0.13392 (15)	0.62543 (7)	0.0199 (5)	
C5	0.69247 (9)	−0.01819 (17)	0.69731 (8)	0.0286 (6)	
H5	0.718560	−0.000389	0.700051	0.034*	
C6	0.68533 (10)	−0.09785 (19)	0.70232 (10)	0.0383 (7)	
H6	0.706606	−0.134781	0.708309	0.046*	
C7	0.64748 (10)	−0.12359 (19)	0.69863 (9)	0.0351 (7)	
H7	0.642816	−0.178275	0.701809	0.042*	
C8	0.61627 (9)	−0.0704 (2)	0.69033 (8)	0.0339 (7)	
H8	0.590331	−0.088352	0.688111	0.041*	
C9	0.62297 (8)	0.00919 (18)	0.68528 (8)	0.0281 (6)	
H9	0.601674	0.046017	0.679787	0.034*	
C10	0.66109 (8)	0.03521 (16)	0.68826 (7)	0.0216 (5)	
C11	0.73849 (8)	0.20598 (17)	0.71715 (7)	0.0252 (5)	
H11	0.723835	0.227806	0.731969	0.030*	
C12	0.77862 (9)	0.22274 (18)	0.72581 (8)	0.0291 (6)	
H12	0.791258	0.256198	0.746513	0.035*	
C13	0.80025 (8)	0.19111 (18)	0.70455 (8)	0.0283 (6)	
H13	0.827744	0.202339	0.710860	0.034*	
C14	0.78189 (8)	0.14287 (18)	0.67393 (8)	0.0284 (6)	
H14	0.796798	0.121024	0.659349	0.034*	
C15	0.74165 (8)	0.12674 (17)	0.66474 (8)	0.0254 (5)	
H15	0.728909	0.094904	0.643450	0.030*	
C16	0.71974 (8)	0.15728 (15)	0.68679 (7)	0.0207 (5)	
C17	0.59326 (9)	0.22703 (18)	0.46934 (8)	0.0285 (6)	
H17	0.582411	0.266718	0.481157	0.034*	
C18	0.57293 (10)	0.2032 (2)	0.43291 (8)	0.0357 (7)	
H18	0.548284	0.227199	0.419692	0.043*	
C19	0.58839 (10)	0.1450 (2)	0.41589 (8)	0.0381 (7)	
H19	0.574448	0.129297	0.390885	0.046*	
C20	0.62419 (10)	0.1091 (2)	0.43504 (9)	0.0363 (7)	
H20	0.634467	0.068274	0.423352	0.044*	
C21	0.64506 (8)	0.13312 (18)	0.47146 (8)	0.0272 (6)	
H21	0.669697	0.109002	0.484572	0.033*	
C22	0.62974 (8)	0.19236 (15)	0.48854 (7)	0.0217 (5)	
C23	0.73408 (8)	0.27716 (17)	0.54350 (8)	0.0251 (5)	
H23	0.723274	0.327613	0.534432	0.030*	
C24	0.77500 (8)	0.26600 (19)	0.55575 (8)	0.0293 (6)	
H24	0.792022	0.308983	0.555024	0.035*	
C25	0.79094 (8)	0.19276 (18)	0.56894 (8)	0.0274 (6)	
H25	0.818864	0.185823	0.577761	0.033*	
C26	0.76630 (9)	0.12963 (18)	0.56932 (8)	0.0298 (6)	
H26	0.777308	0.078982	0.577745	0.036*	
C27	0.72539 (8)	0.13996 (17)	0.55739 (8)	0.0280 (6)	
H27	0.708539	0.096561	0.557950	0.034*	
C28	0.70910 (8)	0.21432 (16)	0.54458 (7)	0.0209 (5)	
C29	0.64600 (7)	0.48370 (15)	0.63747 (7)	0.0187 (5)	
C30	0.62744 (8)	0.55029 (15)	0.61723 (7)	0.0207 (5)	
H30	0.639738	0.598939	0.614471	0.025*	
C31	0.58721 (7)	0.53140 (15)	0.60181 (7)	0.0207 (5)	
H31	0.566765	0.564300	0.586446	0.025*	
C32	0.58342 (7)	0.45436 (15)	0.61368 (7)	0.0180 (5)	
C33	0.48862 (8)	0.44384 (17)	0.54031 (7)	0.0244 (5)	
H33	0.501410	0.404674	0.530060	0.029*	
C34	0.45888 (8)	0.49086 (19)	0.51679 (8)	0.0286 (6)	
H34	0.451096	0.483793	0.490363	0.034*	
C35	0.44069 (9)	0.5481 (2)	0.53214 (9)	0.0331 (6)	
H35	0.420807	0.580836	0.515932	0.040*	
C36	0.45087 (9)	0.55856 (19)	0.57072 (9)	0.0328 (6)	
H36	0.437774	0.597472	0.580799	0.039*	
C37	0.48042 (8)	0.51147 (18)	0.59439 (8)	0.0272 (6)	
H37	0.487587	0.517889	0.620822	0.033*	
C38	0.49958 (7)	0.45463 (16)	0.57919 (7)	0.0208 (5)	
C39	0.51988 (9)	0.32538 (19)	0.66717 (8)	0.0303 (6)	
H39	0.509549	0.282205	0.650688	0.036*	
C40	0.51869 (10)	0.3237 (2)	0.70385 (9)	0.0369 (7)	
H40	0.507105	0.279578	0.712211	0.044*	
C41	0.53423 (9)	0.3856 (2)	0.72812 (8)	0.0336 (7)	
H41	0.533996	0.383280	0.753306	0.040*	
C42	0.55002 (9)	0.4506 (2)	0.71596 (8)	0.0344 (7)	
H42	0.560118	0.493661	0.732594	0.041*	
C43	0.55129 (9)	0.45361 (19)	0.67937 (8)	0.0301 (6)	
H43	0.562384	0.498514	0.671098	0.036*	
C44	0.53624 (8)	0.39055 (17)	0.65478 (7)	0.0242 (5)	
C45	0.72439 (8)	0.58532 (17)	0.62850 (8)	0.0261 (5)	
H45	0.721116	0.543939	0.610590	0.031*	
C46	0.73694 (9)	0.65986 (19)	0.62122 (8)	0.0307 (6)	
H46	0.742408	0.669484	0.598448	0.037*	
C47	0.74146 (10)	0.72017 (18)	0.64735 (9)	0.0322 (6)	
H47	0.749317	0.771656	0.642173	0.039*	
C48	0.73457 (11)	0.70564 (19)	0.68087 (9)	0.0365 (7)	
H48	0.738268	0.746944	0.698882	0.044*	
C49	0.72224 (9)	0.63090 (17)	0.68850 (8)	0.0284 (6)	
H49	0.717732	0.621059	0.711702	0.034*	
C50	0.71657 (7)	0.57093 (15)	0.66204 (7)	0.0204 (5)	
C51	0.66454 (9)	0.51499 (17)	0.72383 (8)	0.0273 (6)	
H51	0.650772	0.555897	0.707433	0.033*	
C52	0.65837 (9)	0.50400 (19)	0.75825 (8)	0.0298 (6)	
H52	0.641124	0.538477	0.765709	0.036*	
C53	0.67740 (9)	0.4428 (2)	0.78153 (8)	0.0334 (7)	
H53	0.673109	0.434881	0.804958	0.040*	
C54	0.70266 (10)	0.3933 (2)	0.77068 (9)	0.0433 (8)	
H54	0.715377	0.350845	0.786617	0.052*	
C55	0.70978 (9)	0.40474 (19)	0.73662 (8)	0.0297 (6)	
H55	0.727435	0.370685	0.729501	0.036*	
C56	0.69089 (7)	0.46614 (16)	0.71331 (7)	0.0203 (5)	
C57	0.61494 (9)	0.24971 (19)	0.73738 (8)	0.0319 (6)	
H57	0.599897	0.285766	0.718866	0.038*	
C58	0.60976 (10)	0.2486 (2)	0.77254 (8)	0.0356 (7)	
H58	0.591299	0.284006	0.777565	0.043*	
C59	0.63101 (9)	0.19681 (18)	0.80041 (8)	0.0288 (6)	
C60	0.62564 (11)	0.1951 (2)	0.83901 (8)	0.0365 (7)	
H60	0.636795	0.143669	0.851106	0.044*	
C61	0.58252 (13)	0.1981 (3)	0.83743 (11)	0.0583 (11)	
H61A	0.580793	0.191356	0.862757	0.087*	
H61B	0.567817	0.155416	0.821195	0.087*	
H61C	0.571129	0.249534	0.827316	0.087*	
C62	0.64956 (13)	0.2617 (2)	0.86351 (9)	0.0469 (9)	
H62A	0.639473	0.313174	0.852359	0.070*	
H62B	0.677491	0.256476	0.865197	0.070*	
H62C	0.647080	0.258149	0.888687	0.070*	
C63	0.65875 (9)	0.14821 (17)	0.79234 (8)	0.0281 (6)	
H63	0.674270	0.113386	0.811201	0.034*	
C64	0.66447 (9)	0.14909 (18)	0.75735 (7)	0.0277 (6)	
H64	0.683923	0.115574	0.752821	0.033*	
C65	0.64170 (8)	0.19908 (16)	0.72881 (7)	0.0225 (5)	
C66	0.77827 (8)	0.35279 (17)	0.64517 (8)	0.0253 (5)	
H66	0.762124	0.323276	0.624376	0.030*	
C67	0.81942 (9)	0.34801 (18)	0.65475 (9)	0.0315 (6)	
H67	0.830802	0.315128	0.640370	0.038*	
C68	0.84426 (8)	0.39064 (18)	0.68512 (9)	0.0312 (6)	
C69	0.88940 (9)	0.3911 (2)	0.69581 (11)	0.0416 (8)	
H69	0.899947	0.397379	0.723685	0.050*	
C70	0.90678 (12)	0.3158 (2)	0.68649 (14)	0.0553 (10)	
H70A	0.897629	0.307958	0.659238	0.083*	
H70B	0.898312	0.270826	0.698631	0.083*	
H70C	0.935752	0.319457	0.695504	0.083*	
C71	0.90273 (9)	0.4639 (2)	0.67887 (10)	0.0397 (7)	
H71A	0.931605	0.462998	0.684940	0.060*	
H71B	0.894807	0.512116	0.689229	0.060*	
H71C	0.890328	0.463334	0.651562	0.060*	
C72	0.82632 (8)	0.43660 (18)	0.70559 (8)	0.0295 (6)	
H72	0.842483	0.465601	0.726566	0.035*	
C73	0.78547 (8)	0.44168 (17)	0.69647 (8)	0.0255 (5)	
H73	0.774234	0.473656	0.711297	0.031*	
C74	0.76058 (7)	0.40018 (16)	0.66563 (7)	0.0209 (5)	
C75	0.65573 (8)	0.38778 (16)	0.48858 (7)	0.0228 (5)	
H75	0.657132	0.338826	0.476494	0.027*	
C76	0.65829 (8)	0.45896 (17)	0.47074 (7)	0.0249 (5)	
H76	0.661028	0.457840	0.446420	0.030*	
C77	0.65694 (8)	0.53147 (17)	0.48770 (8)	0.0266 (6)	
C78	0.65722 (10)	0.60829 (18)	0.46641 (9)	0.0352 (7)	
H78	0.667635	0.595694	0.445287	0.042*	
C79	0.61461 (14)	0.6391 (3)	0.44920 (13)	0.0638 (13)	
H79A	0.598018	0.597233	0.433940	0.096*	
H79B	0.614607	0.685397	0.433375	0.096*	
H79C	0.604059	0.654448	0.469321	0.096*	
C80	0.68402 (18)	0.6711 (2)	0.49064 (14)	0.0681 (14)	
H80A	0.674202	0.685446	0.511366	0.102*	
H80B	0.684141	0.718317	0.475423	0.102*	
H80C	0.711007	0.650140	0.500776	0.102*	
C81	0.65341 (9)	0.53110 (17)	0.52371 (8)	0.0268 (6)	
H81	0.652938	0.580121	0.536069	0.032*	
C82	0.65057 (8)	0.46043 (16)	0.54175 (7)	0.0234 (5)	
H82	0.648241	0.461827	0.566228	0.028*	
C83	0.65108 (7)	0.38766 (16)	0.52441 (7)	0.0193 (5)	
C84	0.48032 (8)	0.25734 (17)	0.57821 (7)	0.0237 (5)	
H84	0.471272	0.304333	0.586868	0.028*	
C85	0.45464 (8)	0.19402 (16)	0.56572 (7)	0.0236 (5)	
H85	0.428232	0.198619	0.566248	0.028*	
C86	0.46608 (8)	0.12385 (17)	0.55240 (7)	0.0240 (5)	
C87	0.43570 (8)	0.05812 (18)	0.53778 (8)	0.0276 (6)	
H87	0.423664	0.045815	0.557825	0.033*	
C88	0.40229 (10)	0.0867 (2)	0.50302 (9)	0.0381 (7)	
H88A	0.390739	0.135612	0.509098	0.057*	
H88B	0.381771	0.045628	0.495308	0.057*	
H88C	0.413059	0.096946	0.482494	0.057*	
C89	0.45343 (10)	−0.01822 (18)	0.52862 (9)	0.0343 (7)	
H89A	0.463516	−0.008939	0.507572	0.051*	
H89B	0.433008	−0.059559	0.521786	0.051*	
H89C	0.475206	−0.035415	0.550614	0.051*	
C90	0.50519 (8)	0.11893 (17)	0.55296 (8)	0.0266 (6)	
H90	0.514086	0.071591	0.544442	0.032*	
C91	0.53183 (8)	0.18147 (17)	0.56564 (8)	0.0267 (6)	
H91	0.558426	0.175825	0.565802	0.032*	
C92	0.51981 (7)	0.25232 (16)	0.57812 (7)	0.0213 (5)	
S1	0.61984 (2)	0.57672 (5)	0.84904 (2)	0.03152 (15)	
F1	0.58461 (9)	0.45029 (15)	0.86472 (7)	0.0650 (7)	
F2	0.59594 (6)	0.44420 (13)	0.81201 (6)	0.0467 (5)	
F3	0.54882 (6)	0.51749 (17)	0.81756 (9)	0.0735 (8)	
O1	0.65694 (7)	0.53792 (17)	0.86767 (7)	0.0482 (6)	
O2	0.60400 (9)	0.62321 (16)	0.87305 (7)	0.0496 (6)	
O3	0.61747 (8)	0.61245 (15)	0.81376 (7)	0.0419 (5)	
C93	0.58557 (10)	0.4933 (2)	0.83522 (10)	0.0381 (7)	
C1A	0.5809 (8)	0.7195 (18)	0.5496 (8)	0.055 (5)	0.44 (4)
H	0.592240	0.675369	0.540967	0.066*	0.44 (4)
C2A	0.6028 (5)	0.7596 (12)	0.5816 (7)	0.044 (4)	0.44 (4)
HA	0.629203	0.743285	0.594455	0.053*	0.44 (4)
C3A	0.5872 (7)	0.8215 (10)	0.5947 (4)	0.040 (4)	0.44 (4)
HB	0.602454	0.849404	0.616461	0.048*	0.44 (4)
C4A	0.5485 (7)	0.8437 (13)	0.5761 (6)	0.044 (4)	0.44 (4)
HC	0.536757	0.885164	0.586031	0.053*	0.44 (4)
C5A	0.5265 (5)	0.8064 (17)	0.5430 (7)	0.048 (4)	0.44 (4)
HD	0.500559	0.824484	0.529527	0.057*	0.44 (4)
C6A	0.5427 (7)	0.7432 (14)	0.5300 (5)	0.049 (5)	0.44 (4)
HE	0.527880	0.715920	0.507874	0.059*	0.44 (4)
C1B	0.5775 (5)	0.7190 (13)	0.5405 (6)	0.045 (3)	0.56 (4)
HF	0.582900	0.677977	0.525545	0.054*	0.56 (4)
C2B	0.6070 (4)	0.7469 (12)	0.5714 (5)	0.050 (3)	0.56 (4)
HG	0.632948	0.725387	0.577434	0.059*	0.56 (4)
C3B	0.5994 (6)	0.8051 (14)	0.5934 (4)	0.057 (4)	0.56 (4)
HH	0.619776	0.823187	0.614991	0.068*	0.56 (4)
C4B	0.5622 (7)	0.8374 (11)	0.5842 (6)	0.055 (4)	0.56 (4)
HI	0.557056	0.878750	0.599207	0.066*	0.56 (4)
C5B	0.5317 (5)	0.8104 (11)	0.5530 (6)	0.049 (4)	0.56 (4)
HJ	0.505911	0.832613	0.546790	0.059*	0.56 (4)
C6B	0.5398 (5)	0.7511 (12)	0.5315 (5)	0.051 (4)	0.56 (4)
HK	0.519421	0.731942	0.510230	0.061*	0.56 (4)
C1C	0.7594 (7)	−0.1583 (8)	0.5750 (4)	0.038 (3)	0.47 (3)
HL	0.775574	−0.190795	0.565157	0.045*	0.47 (3)
C2C	0.7767 (5)	−0.1007 (11)	0.6019 (5)	0.037 (3)	0.47 (3)
HM	0.804499	−0.092427	0.609981	0.045*	0.47 (3)
C3C	0.7526 (6)	−0.0558 (15)	0.6167 (7)	0.047 (5)	0.47 (3)
HN	0.763963	−0.016679	0.635234	0.056*	0.47 (3)
C4C	0.7119 (6)	−0.0676 (19)	0.6046 (9)	0.050 (5)	0.47 (3)
HO	0.695528	−0.036892	0.615012	0.060*	0.47 (3)
C5C	0.6950 (5)	−0.1242 (14)	0.5773 (5)	0.048 (4)	0.47 (3)
HP	0.667163	−0.132485	0.569002	0.057*	0.47 (3)
C6C	0.7188 (7)	−0.1681 (9)	0.5626 (3)	0.046 (4)	0.47 (3)
HQ	0.707246	−0.205813	0.543432	0.055*	0.47 (3)
C1D	0.7403 (7)	−0.1632 (7)	0.5669 (4)	0.048 (4)	0.53 (3)
HR	0.750053	−0.199903	0.552773	0.058*	0.53 (3)
C2D	0.7663 (5)	−0.1123 (10)	0.5917 (5)	0.041 (3)	0.53 (3)
HS	0.793671	−0.113221	0.593994	0.049*	0.53 (3)
C3D	0.7527 (4)	−0.0601 (9)	0.6133 (6)	0.035 (4)	0.53 (3)
HT	0.770535	−0.026161	0.630917	0.042*	0.53 (3)
C4D	0.7122 (4)	−0.0581 (15)	0.6087 (7)	0.036 (3)	0.53 (3)
HU	0.702446	−0.022450	0.623249	0.043*	0.53 (3)
C5D	0.6861 (4)	−0.1082 (8)	0.5829 (4)	0.035 (2)	0.53 (3)
HV	0.658466	−0.105816	0.579422	0.042*	0.53 (3)
C6D	0.7004 (7)	−0.1614 (6)	0.5623 (3)	0.043 (3)	0.53 (3)
HW	0.682790	−0.196538	0.545131	0.051*	0.53 (3)
C1E	0.5025 (5)	−0.0079 (6)	0.6449 (2)	0.063 (2)	0.775 (16)
HX	0.491102	−0.021372	0.619139	0.075*	0.775 (16)
C2E	0.5140 (2)	0.0696 (5)	0.65423 (19)	0.063 (2)	0.775 (16)
HY	0.511363	0.108414	0.635231	0.075*	0.775 (16)
C3E	0.5298 (2)	0.0902 (6)	0.6923 (2)	0.070 (2)	0.775 (16)
HZ	0.538141	0.142852	0.699919	0.083*	0.775 (16)
C4E	0.5326 (2)	0.0285 (8)	0.71890 (15)	0.069 (2)	0.775 (16)
H1	0.542541	0.041920	0.744764	0.083*	0.775 (16)
C5E	0.5219 (3)	−0.0498 (7)	0.7096 (3)	0.071 (3)	0.775 (16)
H4	0.524662	−0.089659	0.728147	0.085*	0.775 (16)
C6E	0.5068 (2)	−0.0662 (6)	0.6714 (3)	0.071 (2)	0.775 (16)
H10	0.499321	−0.119131	0.663415	0.086*	0.775 (16)
C1F	0.505 (2)	0.0065 (18)	0.6426 (8)	0.065 (8)	0.225 (16)
H16	0.497772	0.015412	0.616351	0.078*	0.225 (16)
C2F	0.5131 (13)	0.0713 (16)	0.6656 (10)	0.083 (8)	0.225 (16)
H22	0.509127	0.123261	0.655284	0.100*	0.225 (16)
C3F	0.5274 (11)	0.0607 (17)	0.7048 (10)	0.080 (7)	0.225 (16)
H28	0.537378	0.103174	0.721851	0.095*	0.225 (16)
C4F	0.5258 (11)	−0.018 (2)	0.7164 (7)	0.061 (7)	0.225 (16)
H29	0.532468	−0.025882	0.742713	0.073*	0.225 (16)
C5F	0.5155 (8)	−0.0855 (16)	0.6939 (9)	0.068 (6)	0.225 (16)
H32	0.514760	−0.137056	0.703858	0.082*	0.225 (16)
C6F	0.5061 (7)	−0.0707 (15)	0.6547 (8)	0.058 (6)	0.225 (16)
H38	0.500833	−0.113462	0.637346	0.070*	0.225 (16)
C1G	0.53337 (14)	0.6897 (2)	0.64800 (11)	0.0506 (9)	
H44	0.526870	0.695496	0.621592	0.061*	
C2G	0.57257 (13)	0.6905 (2)	0.67037 (12)	0.0510 (9)	
H50	0.592927	0.696626	0.659268	0.061*	
C3G	0.58228 (13)	0.6825 (2)	0.70879 (11)	0.0504 (9)	
H56	0.609254	0.683619	0.724098	0.061*	
C4G	0.55277 (14)	0.6729 (3)	0.72494 (11)	0.0577 (11)	
H59	0.559353	0.666929	0.751352	0.069*	
C5G	0.51326 (14)	0.6719 (3)	0.70229 (12)	0.0619 (12)	
H61	0.492866	0.665420	0.713310	0.074*	
C6G	0.50365 (13)	0.6804 (3)	0.66386 (12)	0.0539 (10)	
H62	0.476702	0.679843	0.648456	0.065*	
C1H	0.80209 (12)	0.5319 (2)	0.58835 (10)	0.0441 (8)	
H65	0.809427	0.530752	0.614951	0.053*	
C2H	0.82146 (12)	0.5830 (2)	0.57055 (10)	0.0488 (9)	
H68	0.842135	0.616491	0.585064	0.059*	
C3H	0.81055 (12)	0.5846 (2)	0.53200 (10)	0.0461 (8)	
H70	0.823525	0.619620	0.519880	0.055*	
C4H	0.78057 (12)	0.5351 (2)	0.51079 (10)	0.0445 (8)	
H71	0.773215	0.535864	0.484183	0.053*	
C5H	0.76163 (10)	0.4852 (2)	0.52822 (10)	0.0415 (8)	
H74	0.741026	0.451513	0.513717	0.050*	
C6H	0.77253 (10)	0.4838 (2)	0.56717 (10)	0.0382 (7)	
H77	0.759301	0.448997	0.579162	0.046*	

### Atomic displacement parameters (Å^2^)

**Table:**

	*U*^11^	*U*^22^	*U*^33^	*U*^12^	*U*^13^	*U*^23^
Ag1	0.01718 (9)	0.02363 (10)	0.02077 (9)	0.00023 (7)	0.00680 (7)	−0.00063 (6)
Ag2	0.01861 (9)	0.01960 (9)	0.01870 (9)	−0.00177 (6)	0.00486 (6)	−0.00239 (6)
Ag3	0.01932 (9)	0.02002 (9)	0.01916 (9)	0.00257 (6)	0.00563 (7)	0.00374 (6)
P1	0.0172 (3)	0.0211 (3)	0.0153 (3)	0.0005 (2)	0.0040 (2)	−0.0003 (2)
P2	0.0194 (3)	0.0194 (3)	0.0162 (3)	0.0017 (2)	0.0042 (2)	0.0016 (2)
P3	0.0160 (3)	0.0184 (3)	0.0160 (3)	−0.0006 (2)	0.0029 (2)	−0.0009 (2)
P4	0.0142 (3)	0.0223 (3)	0.0173 (3)	0.0006 (2)	0.0035 (2)	0.0006 (2)
N1	0.0160 (10)	0.0226 (11)	0.0244 (11)	0.0007 (8)	0.0074 (8)	0.0000 (8)
N2	0.0184 (10)	0.0235 (11)	0.0177 (10)	0.0009 (8)	0.0073 (8)	0.0001 (8)
N3	0.0159 (9)	0.0199 (10)	0.0196 (10)	−0.0012 (8)	0.0025 (8)	−0.0038 (8)
N4	0.0193 (10)	0.0196 (10)	0.0166 (10)	0.0026 (8)	0.0029 (8)	0.0015 (8)
N5	0.0244 (11)	0.0211 (10)	0.0178 (10)	0.0039 (8)	0.0082 (8)	0.0046 (8)
N6	0.0183 (10)	0.0183 (10)	0.0150 (9)	0.0004 (8)	0.0028 (7)	0.0013 (7)
C1	0.0202 (11)	0.0217 (12)	0.0158 (11)	0.0002 (10)	0.0051 (9)	−0.0013 (9)
C2	0.0228 (12)	0.0223 (12)	0.0190 (11)	−0.0022 (10)	0.0031 (9)	−0.0007 (9)
C3	0.0220 (12)	0.0227 (13)	0.0220 (12)	−0.0028 (10)	0.0059 (10)	0.0001 (10)
C4	0.0189 (11)	0.0197 (12)	0.0203 (11)	0.0033 (9)	0.0052 (9)	0.0027 (9)
C5	0.0242 (13)	0.0241 (14)	0.0365 (15)	0.0005 (11)	0.0082 (11)	0.0056 (11)
C6	0.0364 (17)	0.0264 (15)	0.0492 (19)	0.0021 (13)	0.0097 (14)	0.0078 (13)
C7	0.0398 (17)	0.0261 (15)	0.0362 (16)	−0.0071 (13)	0.0074 (13)	0.0049 (12)
C8	0.0284 (14)	0.0388 (17)	0.0300 (14)	−0.0108 (13)	0.0030 (11)	0.0063 (12)
C9	0.0236 (13)	0.0323 (15)	0.0266 (13)	0.0017 (11)	0.0054 (10)	0.0065 (11)
C10	0.0230 (12)	0.0227 (12)	0.0176 (11)	−0.0001 (10)	0.0043 (9)	0.0025 (9)
C11	0.0280 (14)	0.0280 (14)	0.0188 (12)	−0.0009 (11)	0.0062 (10)	0.0004 (10)
C12	0.0297 (14)	0.0316 (15)	0.0223 (12)	−0.0075 (12)	0.0031 (11)	0.0001 (11)
C13	0.0209 (13)	0.0329 (15)	0.0284 (14)	−0.0026 (11)	0.0038 (11)	0.0059 (11)
C14	0.0230 (13)	0.0319 (15)	0.0316 (14)	0.0010 (11)	0.0105 (11)	0.0006 (11)
C15	0.0243 (13)	0.0250 (13)	0.0255 (13)	0.0009 (10)	0.0060 (10)	−0.0018 (10)
C16	0.0211 (12)	0.0208 (12)	0.0182 (11)	0.0008 (10)	0.0035 (9)	0.0029 (9)
C17	0.0277 (14)	0.0298 (14)	0.0245 (13)	0.0020 (11)	0.0036 (11)	−0.0007 (11)
C18	0.0318 (15)	0.0429 (18)	0.0236 (14)	−0.0027 (13)	−0.0034 (12)	0.0020 (12)
C19	0.0391 (17)	0.054 (2)	0.0188 (13)	−0.0151 (15)	0.0060 (12)	−0.0095 (13)
C20	0.0362 (16)	0.0448 (18)	0.0301 (15)	−0.0093 (14)	0.0138 (13)	−0.0157 (13)
C21	0.0251 (13)	0.0327 (15)	0.0246 (13)	−0.0030 (11)	0.0091 (10)	−0.0069 (11)
C22	0.0244 (13)	0.0228 (12)	0.0173 (11)	−0.0040 (10)	0.0058 (10)	−0.0005 (9)
C23	0.0229 (13)	0.0255 (13)	0.0275 (13)	0.0029 (10)	0.0089 (10)	0.0021 (10)
C24	0.0228 (13)	0.0342 (15)	0.0323 (14)	−0.0025 (11)	0.0111 (11)	−0.0005 (12)
C25	0.0197 (12)	0.0393 (16)	0.0229 (13)	0.0065 (11)	0.0063 (10)	−0.0007 (11)
C26	0.0257 (14)	0.0304 (15)	0.0324 (14)	0.0073 (11)	0.0078 (11)	0.0026 (12)
C27	0.0256 (13)	0.0254 (14)	0.0323 (14)	0.0031 (11)	0.0083 (11)	0.0043 (11)
C28	0.0205 (12)	0.0264 (13)	0.0158 (11)	0.0021 (10)	0.0059 (9)	−0.0013 (9)
C29	0.0161 (11)	0.0221 (12)	0.0177 (11)	0.0003 (9)	0.0050 (9)	−0.0010 (9)
C30	0.0209 (12)	0.0190 (12)	0.0212 (11)	−0.0010 (10)	0.0053 (9)	−0.0003 (9)
C31	0.0201 (12)	0.0207 (12)	0.0186 (11)	0.0044 (10)	0.0023 (9)	0.0020 (9)
C32	0.0154 (11)	0.0204 (12)	0.0167 (11)	0.0052 (9)	0.0029 (9)	0.0004 (9)
C33	0.0190 (12)	0.0315 (14)	0.0213 (12)	0.0012 (10)	0.0045 (9)	0.0007 (10)
C34	0.0210 (12)	0.0386 (16)	0.0226 (13)	0.0001 (11)	0.0018 (10)	0.0043 (11)
C35	0.0221 (13)	0.0384 (17)	0.0335 (15)	0.0048 (12)	0.0015 (11)	0.0065 (13)
C36	0.0245 (13)	0.0364 (16)	0.0364 (15)	0.0075 (12)	0.0081 (12)	−0.0022 (13)
C37	0.0211 (12)	0.0354 (15)	0.0250 (13)	0.0040 (11)	0.0071 (10)	−0.0016 (11)
C38	0.0153 (11)	0.0256 (13)	0.0198 (11)	−0.0013 (10)	0.0034 (9)	0.0009 (10)
C39	0.0270 (14)	0.0380 (16)	0.0253 (13)	−0.0060 (12)	0.0075 (11)	0.0019 (12)
C40	0.0307 (15)	0.051 (2)	0.0306 (15)	−0.0027 (14)	0.0125 (12)	0.0083 (13)
C41	0.0245 (13)	0.055 (2)	0.0229 (13)	0.0026 (13)	0.0093 (11)	0.0034 (13)
C42	0.0304 (15)	0.0477 (18)	0.0243 (13)	0.0007 (13)	0.0078 (11)	−0.0044 (13)
C43	0.0283 (14)	0.0374 (16)	0.0235 (13)	0.0011 (12)	0.0068 (11)	−0.0025 (11)
C44	0.0184 (11)	0.0337 (14)	0.0193 (12)	0.0027 (11)	0.0042 (9)	0.0027 (10)
C45	0.0274 (13)	0.0262 (14)	0.0261 (13)	−0.0041 (11)	0.0103 (11)	−0.0039 (11)
C46	0.0343 (15)	0.0330 (15)	0.0268 (13)	−0.0033 (12)	0.0126 (12)	0.0010 (12)
C47	0.0389 (16)	0.0226 (14)	0.0357 (15)	−0.0081 (12)	0.0126 (13)	0.0017 (12)
C48	0.052 (2)	0.0268 (15)	0.0319 (15)	−0.0096 (14)	0.0151 (14)	−0.0066 (12)
C49	0.0367 (15)	0.0250 (14)	0.0239 (13)	−0.0064 (12)	0.0102 (11)	−0.0030 (10)
C50	0.0188 (11)	0.0207 (12)	0.0191 (11)	−0.0005 (9)	0.0025 (9)	0.0009 (9)
C51	0.0326 (14)	0.0262 (14)	0.0242 (13)	0.0046 (11)	0.0107 (11)	0.0014 (10)
C52	0.0288 (14)	0.0368 (16)	0.0257 (13)	−0.0016 (12)	0.0116 (11)	−0.0045 (11)
C53	0.0285 (14)	0.0508 (19)	0.0211 (13)	−0.0020 (13)	0.0082 (11)	0.0070 (12)
C54	0.0368 (17)	0.060 (2)	0.0373 (17)	0.0176 (16)	0.0173 (14)	0.0257 (16)
C55	0.0259 (13)	0.0359 (16)	0.0285 (14)	0.0082 (12)	0.0104 (11)	0.0102 (12)
C56	0.0181 (11)	0.0237 (12)	0.0177 (11)	−0.0008 (10)	0.0035 (9)	−0.0002 (9)
C57	0.0363 (15)	0.0366 (16)	0.0232 (13)	0.0126 (13)	0.0102 (11)	0.0076 (12)
C58	0.0402 (17)	0.0430 (18)	0.0286 (14)	0.0125 (14)	0.0179 (13)	0.0047 (13)
C59	0.0321 (15)	0.0330 (15)	0.0220 (13)	0.0032 (12)	0.0098 (11)	0.0021 (11)
C60	0.0473 (19)	0.0414 (18)	0.0244 (14)	0.0116 (14)	0.0164 (13)	0.0063 (12)
C61	0.055 (2)	0.092 (3)	0.0368 (19)	0.011 (2)	0.0282 (18)	0.008 (2)
C62	0.064 (2)	0.048 (2)	0.0296 (16)	0.0103 (18)	0.0167 (15)	−0.0014 (14)
C63	0.0330 (14)	0.0279 (14)	0.0221 (12)	0.0071 (12)	0.0070 (11)	0.0047 (10)
C64	0.0308 (14)	0.0299 (14)	0.0219 (12)	0.0048 (12)	0.0078 (11)	0.0011 (11)
C65	0.0251 (13)	0.0223 (12)	0.0197 (12)	0.0020 (10)	0.0065 (10)	0.0040 (9)
C66	0.0219 (12)	0.0295 (14)	0.0256 (13)	0.0015 (11)	0.0090 (10)	0.0014 (11)
C67	0.0252 (14)	0.0314 (15)	0.0428 (16)	0.0028 (12)	0.0177 (12)	0.0025 (13)
C68	0.0186 (12)	0.0314 (15)	0.0430 (16)	0.0009 (11)	0.0088 (11)	0.0072 (12)
C69	0.0187 (13)	0.0465 (19)	0.057 (2)	0.0037 (13)	0.0080 (13)	0.0089 (16)
C70	0.0331 (18)	0.052 (2)	0.080 (3)	0.0078 (16)	0.0159 (19)	0.011 (2)
C71	0.0226 (14)	0.0430 (19)	0.054 (2)	−0.0036 (13)	0.0129 (13)	−0.0019 (15)
C72	0.0181 (12)	0.0321 (15)	0.0332 (14)	−0.0034 (11)	0.0008 (11)	0.0026 (12)
C73	0.0209 (12)	0.0277 (14)	0.0263 (13)	−0.0004 (10)	0.0051 (10)	−0.0003 (11)
C74	0.0171 (11)	0.0222 (12)	0.0217 (12)	0.0007 (9)	0.0037 (9)	0.0038 (9)
C75	0.0235 (12)	0.0245 (13)	0.0203 (12)	0.0032 (10)	0.0070 (10)	−0.0009 (10)
C76	0.0253 (13)	0.0310 (14)	0.0208 (12)	0.0034 (11)	0.0108 (10)	0.0034 (10)
C77	0.0259 (13)	0.0272 (14)	0.0294 (14)	0.0048 (11)	0.0127 (11)	0.0054 (11)
C78	0.0469 (18)	0.0289 (15)	0.0407 (16)	0.0096 (13)	0.0295 (14)	0.0102 (13)
C79	0.067 (3)	0.064 (3)	0.073 (3)	0.032 (2)	0.040 (2)	0.047 (2)
C80	0.113 (4)	0.0304 (19)	0.070 (3)	−0.016 (2)	0.042 (3)	0.0024 (19)
C81	0.0324 (14)	0.0217 (13)	0.0281 (13)	0.0018 (11)	0.0121 (11)	−0.0010 (10)
C82	0.0268 (13)	0.0252 (13)	0.0190 (11)	0.0024 (10)	0.0084 (10)	−0.0004 (10)
C83	0.0150 (11)	0.0247 (13)	0.0178 (11)	0.0018 (9)	0.0045 (9)	0.0016 (9)
C84	0.0210 (12)	0.0267 (13)	0.0236 (12)	−0.0001 (10)	0.0074 (10)	−0.0018 (10)
C85	0.0169 (12)	0.0289 (14)	0.0248 (12)	−0.0014 (10)	0.0065 (10)	0.0009 (10)
C86	0.0241 (13)	0.0260 (13)	0.0206 (12)	−0.0047 (10)	0.0053 (10)	−0.0012 (10)
C87	0.0266 (13)	0.0304 (14)	0.0259 (13)	−0.0083 (11)	0.0084 (11)	−0.0020 (11)
C88	0.0306 (15)	0.0359 (17)	0.0388 (17)	−0.0086 (13)	−0.0015 (13)	−0.0015 (13)
C89	0.0351 (16)	0.0285 (15)	0.0362 (16)	−0.0078 (12)	0.0071 (13)	−0.0082 (12)
C90	0.0278 (13)	0.0241 (13)	0.0299 (14)	−0.0034 (11)	0.0121 (11)	−0.0071 (11)
C91	0.0218 (13)	0.0278 (14)	0.0313 (14)	−0.0013 (11)	0.0095 (11)	−0.0032 (11)
C92	0.0180 (11)	0.0244 (13)	0.0212 (11)	−0.0010 (10)	0.0056 (9)	0.0017 (10)
S1	0.0284 (3)	0.0401 (4)	0.0273 (3)	−0.0051 (3)	0.0106 (3)	−0.0030 (3)
F1	0.0924 (19)	0.0536 (14)	0.0709 (15)	−0.0237 (13)	0.0570 (15)	−0.0081 (12)
F2	0.0484 (12)	0.0489 (12)	0.0448 (11)	−0.0060 (9)	0.0177 (9)	−0.0160 (9)
F3	0.0230 (10)	0.0780 (18)	0.110 (2)	−0.0047 (11)	0.0071 (12)	−0.0260 (16)
O1	0.0296 (11)	0.0619 (17)	0.0438 (13)	−0.0030 (11)	−0.0015 (10)	0.0024 (12)
O2	0.0630 (17)	0.0493 (15)	0.0467 (14)	−0.0121 (13)	0.0319 (13)	−0.0150 (11)
O3	0.0492 (14)	0.0436 (13)	0.0374 (12)	0.0022 (11)	0.0202 (10)	0.0053 (10)
C93	0.0333 (16)	0.0444 (18)	0.0410 (17)	−0.0065 (14)	0.0180 (13)	−0.0101 (14)
C1A	0.094 (11)	0.031 (7)	0.047 (11)	0.008 (6)	0.032 (7)	0.014 (6)
C2A	0.047 (6)	0.042 (7)	0.047 (9)	0.013 (5)	0.020 (5)	0.020 (6)
C3A	0.044 (8)	0.038 (6)	0.028 (5)	0.004 (6)	−0.001 (6)	0.004 (4)
C4A	0.042 (8)	0.057 (6)	0.027 (6)	0.021 (6)	0.002 (5)	0.003 (4)
C5A	0.041 (6)	0.069 (9)	0.030 (8)	−0.011 (5)	0.007 (4)	−0.001 (5)
C6A	0.087 (11)	0.040 (8)	0.026 (6)	−0.014 (7)	0.024 (6)	0.005 (5)
C1B	0.071 (7)	0.030 (5)	0.042 (8)	0.006 (4)	0.030 (5)	0.006 (5)
C2B	0.056 (5)	0.057 (7)	0.043 (6)	0.008 (4)	0.026 (4)	0.021 (5)
C3B	0.064 (8)	0.068 (10)	0.041 (4)	0.000 (7)	0.021 (5)	0.005 (5)
C4B	0.066 (10)	0.063 (8)	0.043 (9)	−0.009 (7)	0.026 (7)	−0.015 (7)
C5B	0.054 (7)	0.045 (5)	0.049 (9)	0.012 (5)	0.018 (6)	0.000 (6)
C6B	0.053 (6)	0.044 (7)	0.050 (8)	−0.005 (5)	0.009 (5)	−0.008 (5)
C1C	0.055 (8)	0.038 (5)	0.027 (5)	−0.007 (5)	0.021 (6)	−0.001 (4)
C2C	0.048 (6)	0.031 (5)	0.031 (6)	−0.012 (5)	0.011 (5)	0.001 (4)
C3C	0.052 (8)	0.045 (10)	0.041 (8)	−0.001 (6)	0.010 (6)	−0.010 (7)
C4C	0.052 (8)	0.047 (12)	0.049 (9)	0.009 (6)	0.011 (6)	0.007 (6)
C5C	0.036 (6)	0.050 (11)	0.048 (8)	−0.002 (6)	0.000 (5)	0.014 (6)
C6C	0.057 (9)	0.045 (6)	0.023 (4)	−0.012 (8)	−0.004 (7)	0.001 (3)
C1D	0.067 (10)	0.047 (6)	0.037 (7)	0.014 (8)	0.026 (8)	0.007 (4)
C2D	0.046 (6)	0.037 (7)	0.046 (8)	0.012 (5)	0.022 (5)	0.020 (5)
C3D	0.033 (6)	0.024 (6)	0.042 (7)	−0.002 (4)	0.005 (5)	0.013 (4)
C4D	0.029 (5)	0.027 (5)	0.045 (6)	−0.004 (4)	0.003 (4)	−0.014 (4)
C5D	0.044 (5)	0.017 (4)	0.036 (4)	0.001 (4)	0.001 (4)	−0.006 (3)
C6D	0.064 (8)	0.030 (4)	0.032 (4)	0.007 (5)	0.013 (5)	0.002 (3)
C1E	0.036 (3)	0.108 (6)	0.042 (3)	0.006 (5)	0.008 (3)	0.004 (3)
C2E	0.043 (3)	0.108 (5)	0.035 (3)	0.022 (3)	0.009 (3)	0.004 (3)
C3E	0.056 (3)	0.104 (5)	0.044 (3)	0.034 (3)	0.008 (3)	−0.001 (3)
C4E	0.061 (4)	0.114 (6)	0.033 (3)	0.036 (4)	0.016 (2)	0.010 (3)
C5E	0.043 (4)	0.108 (6)	0.063 (4)	0.022 (4)	0.017 (3)	0.007 (4)
C6E	0.033 (3)	0.120 (5)	0.065 (4)	0.007 (3)	0.022 (3)	0.014 (4)
C1F	0.056 (13)	0.072 (9)	0.064 (10)	0.007 (7)	0.014 (8)	0.012 (6)
C2F	0.085 (12)	0.083 (9)	0.083 (10)	0.013 (8)	0.027 (8)	−0.002 (7)
C3F	0.085 (11)	0.079 (9)	0.085 (9)	0.015 (8)	0.043 (8)	0.009 (7)
C4F	0.056 (10)	0.073 (9)	0.058 (9)	0.012 (8)	0.024 (7)	0.006 (6)
C5F	0.061 (10)	0.081 (9)	0.066 (9)	0.011 (7)	0.025 (7)	0.009 (6)
C6F	0.042 (8)	0.073 (8)	0.062 (8)	0.001 (6)	0.019 (7)	0.002 (7)
C1G	0.067 (3)	0.046 (2)	0.0369 (18)	−0.0030 (18)	0.0136 (18)	−0.0021 (15)
C2G	0.055 (2)	0.050 (2)	0.052 (2)	−0.0103 (18)	0.0233 (19)	−0.0069 (17)
C3G	0.049 (2)	0.049 (2)	0.046 (2)	−0.0054 (17)	0.0046 (17)	−0.0041 (16)
C4G	0.067 (3)	0.068 (3)	0.0358 (19)	−0.015 (2)	0.0129 (18)	−0.0059 (18)
C5G	0.060 (3)	0.084 (3)	0.047 (2)	−0.016 (2)	0.024 (2)	−0.013 (2)
C6G	0.049 (2)	0.061 (3)	0.047 (2)	−0.0019 (19)	0.0080 (17)	−0.0078 (18)
C1H	0.053 (2)	0.047 (2)	0.0344 (17)	0.0003 (17)	0.0179 (15)	−0.0063 (15)
C2H	0.052 (2)	0.050 (2)	0.0421 (19)	−0.0116 (17)	0.0119 (16)	−0.0152 (16)
C3H	0.058 (2)	0.0408 (19)	0.0422 (18)	−0.0105 (17)	0.0194 (16)	−0.0036 (15)
C4H	0.061 (2)	0.0339 (17)	0.0327 (16)	−0.0022 (16)	0.0065 (15)	0.0025 (13)
C5H	0.0375 (17)	0.0365 (17)	0.0411 (18)	0.0000 (14)	−0.0009 (14)	0.0061 (14)
C6H	0.0402 (17)	0.0339 (16)	0.0430 (18)	0.0070 (14)	0.0168 (14)	0.0049 (14)

### Geometric parameters (Å, º)

**Table:**

Ag1—Ag2	3.2177 (2)	C68—C72	1.383 (4)
Ag2—Ag3	2.8753 (3)	C69—H69	1.0000
Ag1—Ag3	3.3165 (2)	C69—C70	1.500 (5)
Ag1—N1	2.168 (2)	C69—C71	1.525 (5)
Ag1—N2	2.173 (2)	C70—H70A	0.9800
Ag2—N3	2.127 (2)	C70—H70B	0.9800
Ag2—N4	2.129 (2)	C70—H70C	0.9800
Ag3—N5	2.136 (2)	C71—H71A	0.9800
Ag3—N6	2.130 (2)	C71—H71B	0.9800
P1—N2	1.610 (2)	C71—H71C	0.9800
P1—C1	1.767 (3)	C72—H72	0.9500
P1—C22	1.810 (3)	C72—C73	1.387 (4)
P1—C28	1.814 (3)	C73—H73	0.9500
P2—N5	1.610 (2)	C73—C74	1.401 (4)
P2—C4	1.776 (3)	C75—H75	0.9500
P2—C10	1.811 (3)	C75—C76	1.390 (4)
P2—C16	1.810 (3)	C75—C83	1.408 (3)
P3—N3	1.608 (2)	C76—H76	0.9500
P3—C29	1.771 (2)	C76—C77	1.385 (4)
P3—C50	1.800 (3)	C77—C78	1.523 (4)
P3—C56	1.814 (3)	C77—C81	1.399 (4)
P4—N1	1.610 (2)	C78—H78	1.0000
P4—C32	1.768 (3)	C78—C79	1.538 (5)
P4—C38	1.797 (3)	C78—C80	1.519 (6)
P4—C44	1.814 (3)	C79—H79A	0.9800
N1—C92	1.413 (3)	C79—H79B	0.9800
N2—C83	1.416 (3)	C79—H79C	0.9800
N3—C74	1.425 (3)	C80—H80A	0.9800
N4—C1	1.371 (3)	C80—H80B	0.9800
N4—C4	1.365 (3)	C80—H80C	0.9800
N5—C65	1.421 (3)	C81—H81	0.9500
N6—C29	1.364 (3)	C81—C82	1.389 (4)
N6—C32	1.376 (3)	C82—H82	0.9500
C1—C2	1.393 (4)	C82—C83	1.391 (4)
C2—H2	0.9500	C84—H84	0.9500
C2—C3	1.405 (4)	C84—C85	1.386 (4)
C3—H3	0.9500	C84—C92	1.410 (4)
C3—C4	1.399 (4)	C85—H85	0.9500
C5—H5	0.9500	C85—C86	1.393 (4)
C5—C6	1.389 (4)	C86—C87	1.523 (4)
C5—C10	1.389 (4)	C86—C90	1.389 (4)
C6—H6	0.9500	C87—H87	1.0000
C6—C7	1.380 (5)	C87—C88	1.538 (4)
C7—H7	0.9500	C87—C89	1.519 (4)
C7—C8	1.384 (5)	C88—H88A	0.9800
C8—H8	0.9500	C88—H88B	0.9800
C8—C9	1.384 (4)	C88—H88C	0.9800
C9—H9	0.9500	C89—H89A	0.9800
C9—C10	1.396 (4)	C89—H89B	0.9800
C11—H11	0.9500	C89—H89C	0.9800
C11—C12	1.390 (4)	C90—H90	0.9500
C11—C16	1.391 (4)	C90—C91	1.396 (4)
C12—H12	0.9500	C91—H91	0.9500
C12—C13	1.381 (4)	C91—C92	1.398 (4)
C13—H13	0.9500	S1—O1	1.440 (3)
C13—C14	1.390 (4)	S1—O2	1.439 (3)
C14—H14	0.9500	S1—O3	1.434 (2)
C14—C15	1.390 (4)	S1—C93	1.824 (3)
C15—H15	0.9500	F1—C93	1.334 (4)
C15—C16	1.404 (4)	F2—C93	1.337 (4)
C17—H17	0.9500	F3—C93	1.329 (4)
C17—C18	1.389 (4)	C1A—H	0.9500
C17—C22	1.397 (4)	C1A—C2A	1.383 (14)
C18—H18	0.9500	C1A—C6A	1.384 (14)
C18—C19	1.378 (5)	C2A—HA	0.9500
C19—H19	0.9500	C2A—C3A	1.347 (14)
C19—C20	1.387 (5)	C3A—HB	0.9500
C20—H20	0.9500	C3A—C4A	1.383 (13)
C20—C21	1.394 (4)	C4A—HC	0.9500
C21—H21	0.9500	C4A—C5A	1.391 (13)
C21—C22	1.388 (4)	C5A—HD	0.9500
C23—H23	0.9500	C5A—C6A	1.373 (14)
C23—C24	1.394 (4)	C6A—HE	0.9500
C23—C28	1.391 (4)	C1B—HF	0.9500
C24—H24	0.9500	C1B—C2B	1.377 (12)
C24—C25	1.382 (4)	C1B—C6B	1.383 (11)
C25—H25	0.9500	C2B—HG	0.9500
C25—C26	1.381 (4)	C2B—C3B	1.364 (13)
C26—H26	0.9500	C3B—HH	0.9500
C26—C27	1.392 (4)	C3B—C4B	1.371 (14)
C27—H27	0.9500	C4B—HI	0.9500
C27—C28	1.399 (4)	C4B—C5B	1.396 (13)
C29—C30	1.397 (4)	C5B—HJ	0.9500
C30—H30	0.9500	C5B—C6B	1.371 (11)
C30—C31	1.400 (4)	C6B—HK	0.9500
C31—H31	0.9500	C1C—HL	0.9500
C31—C32	1.392 (4)	C1C—C2C	1.396 (11)
C33—H33	0.9500	C1C—C6C	1.378 (14)
C33—C34	1.391 (4)	C2C—HM	0.9500
C33—C38	1.399 (4)	C2C—C3C	1.386 (12)
C34—H34	0.9500	C3C—HN	0.9500
C34—C35	1.385 (4)	C3C—C4C	1.388 (13)
C35—H35	0.9500	C4C—HO	0.9500
C35—C36	1.390 (4)	C4C—C5C	1.390 (13)
C36—H36	0.9500	C5C—HP	0.9500
C36—C37	1.390 (4)	C5C—C6C	1.368 (15)
C37—H37	0.9500	C6C—HQ	0.9500
C37—C38	1.398 (4)	C1D—HR	0.9500
C39—H39	0.9500	C1D—C2D	1.384 (14)
C39—C40	1.393 (4)	C1D—C6D	1.375 (13)
C39—C44	1.390 (4)	C2D—HS	0.9500
C40—H40	0.9500	C2D—C3D	1.385 (11)
C40—C41	1.380 (5)	C3D—HT	0.9500
C41—H41	0.9500	C3D—C4D	1.395 (10)
C41—C42	1.374 (5)	C4D—HU	0.9500
C42—H42	0.9500	C4D—C5D	1.396 (9)
C42—C43	1.391 (4)	C5D—HV	0.9500
C43—H43	0.9500	C5D—C6D	1.382 (11)
C43—C44	1.398 (4)	C6D—HW	0.9500
C45—H45	0.9500	C1E—HX	0.9500
C45—C46	1.389 (4)	C1E—C2E	1.380 (10)
C45—C50	1.396 (4)	C1E—C6E	1.371 (10)
C46—H46	0.9500	C2E—HY	0.9500
C46—C47	1.386 (4)	C2E—C3E	1.404 (8)
C47—H47	0.9500	C3E—HZ	0.9500
C47—C48	1.380 (4)	C3E—C4E	1.423 (11)
C48—H48	0.9500	C4E—H1	0.9500
C48—C49	1.392 (4)	C4E—C5E	1.386 (13)
C49—H49	0.9500	C5E—H4	0.9500
C49—C50	1.386 (4)	C5E—C6E	1.394 (12)
C51—H51	0.9500	C6E—H10	0.9500
C51—C52	1.392 (4)	C1F—H16	0.9500
C51—C56	1.396 (4)	C1F—C2F	1.366 (18)
C52—H52	0.9500	C1F—C6F	1.374 (18)
C52—C53	1.382 (4)	C2F—H22	0.9500
C53—H53	0.9500	C2F—C3F	1.407 (19)
C53—C54	1.380 (5)	C3F—H28	0.9500
C54—H54	0.9500	C3F—C4F	1.40 (2)
C54—C55	1.395 (4)	C4F—H29	0.9500
C55—H55	0.9500	C4F—C5F	1.40 (2)
C55—C56	1.383 (4)	C5F—H32	0.9500
C57—H57	0.9500	C5F—C6F	1.42 (2)
C57—C58	1.391 (4)	C6F—H38	0.9500
C57—C65	1.391 (4)	C1G—H44	0.9500
C58—H58	0.9500	C1G—C2G	1.381 (6)
C58—C59	1.388 (4)	C1G—C6G	1.379 (6)
C59—C60	1.523 (4)	C2G—H50	0.9500
C59—C63	1.389 (4)	C2G—C3G	1.380 (6)
C60—H60	1.0000	C3G—H56	0.9500
C60—C61	1.518 (5)	C3G—C4G	1.380 (6)
C60—C62	1.527 (5)	C4G—H59	0.9500
C61—H61A	0.9800	C4G—C5G	1.393 (6)
C61—H61B	0.9800	C5G—H61	0.9500
C61—H61C	0.9800	C5G—C6G	1.382 (6)
C62—H62A	0.9800	C6G—H62	0.9500
C62—H62B	0.9800	C1H—H65	0.9500
C62—H62C	0.9800	C1H—C2H	1.398 (6)
C63—H63	0.9500	C1H—C6H	1.366 (5)
C63—C64	1.393 (4)	C2H—H68	0.9500
C64—H64	0.9500	C2H—C3H	1.376 (5)
C64—C65	1.401 (4)	C3H—H70	0.9500
C66—H66	0.9500	C3H—C4H	1.389 (5)
C66—C67	1.395 (4)	C4H—H71	0.9500
C66—C74	1.390 (4)	C4H—C5H	1.368 (5)
C67—H67	0.9500	C5H—H74	0.9500
C67—C68	1.399 (5)	C5H—C6H	1.390 (5)
C68—C69	1.528 (4)	C6H—H77	0.9500
			
Ag1—Ag2—Ag3	65.677 (6)	C67—C66—H66	119.6
Ag2—Ag1—Ag3	52.186 (5)	C74—C66—H66	119.6
Ag2—Ag3—Ag1	62.138 (6)	C74—C66—C67	120.9 (3)
N1—Ag1—N2	173.61 (8)	C66—C67—H67	119.3
N3—Ag2—N4	170.50 (8)	C66—C67—C68	121.4 (3)
N5—Ag3—N6	174.27 (8)	C68—C67—H67	119.3
C1—N4—C4	105.6 (2)	C67—C68—C69	123.8 (3)
C29—N6—C32	105.3 (2)	C72—C68—C67	117.2 (3)
C1—P1—N2	104.88 (12)	C72—C68—C69	119.0 (3)
N2—P1—C22	114.55 (12)	C68—C69—H69	106.8
N2—P1—C28	113.97 (12)	C70—C69—C68	114.3 (3)
C1—P1—C22	108.46 (12)	C70—C69—H69	106.8
C1—P1—C28	104.60 (12)	C70—C69—C71	112.1 (3)
C22—P1—C28	109.64 (12)	C71—C69—C68	109.6 (3)
C4—P2—N5	111.60 (11)	C71—C69—H69	106.8
N5—P2—C10	114.31 (12)	C69—C70—H70A	109.5
N5—P2—C16	113.43 (12)	C69—C70—H70B	109.5
C4—P2—C10	103.05 (12)	C69—C70—H70C	109.5
C4—P2—C16	104.80 (12)	H70A—C70—H70B	109.5
C16—P2—C10	108.74 (12)	H70A—C70—H70C	109.5
C29—P3—N3	111.65 (11)	H70B—C70—H70C	109.5
N3—P3—C50	112.26 (12)	C69—C71—H71A	109.5
N3—P3—C56	115.38 (12)	C69—C71—H71B	109.5
C29—P3—C50	103.33 (12)	C69—C71—H71C	109.5
C29—P3—C56	102.99 (12)	H71A—C71—H71B	109.5
C50—P3—C56	110.19 (12)	H71A—C71—H71C	109.5
C32—P4—N1	105.67 (11)	H71B—C71—H71C	109.5
N1—Ag1—Ag2	121.87 (6)	C68—C72—H72	119.0
N1—Ag1—Ag3	69.92 (6)	C68—C72—C73	122.1 (3)
N2—Ag1—Ag2	64.04 (6)	C73—C72—H72	119.0
N2—Ag1—Ag3	116.14 (6)	C72—C73—H73	119.6
N3—Ag2—Ag1	120.57 (6)	C72—C73—C74	120.8 (3)
N3—Ag2—Ag3	92.63 (6)	C74—C73—H73	119.6
N5—Ag3—Ag1	121.38 (6)	C66—C74—N3	118.5 (2)
N5—Ag3—Ag2	95.83 (6)	C66—C74—C73	117.7 (2)
N4—Ag2—Ag1	65.22 (6)	C73—C74—N3	123.8 (2)
N4—Ag2—Ag3	83.00 (6)	C76—C75—H75	119.7
N6—Ag3—Ag1	61.87 (5)	C76—C75—C83	120.5 (2)
N6—Ag3—Ag2	81.53 (6)	C83—C75—H75	119.7
N1—P4—C38	114.85 (12)	C75—C76—H76	119.3
N1—P4—C44	116.26 (13)	C77—C76—C75	121.4 (2)
C32—P4—C38	106.20 (12)	C77—C76—H76	119.3
C32—P4—C44	104.41 (12)	C76—C77—C78	120.0 (2)
C38—P4—C44	108.40 (12)	C76—C77—C81	117.9 (3)
P4—N1—Ag1	113.10 (12)	C81—C77—C78	122.0 (3)
C92—N1—Ag1	120.07 (17)	C77—C78—H78	107.6
C92—N1—P4	124.86 (18)	C77—C78—C79	109.7 (3)
P1—N2—Ag1	111.44 (11)	C79—C78—H78	107.6
C83—N2—Ag1	119.33 (16)	C80—C78—C77	112.6 (3)
C83—N2—P1	125.50 (18)	C80—C78—H78	107.6
P3—N3—Ag2	122.03 (11)	C80—C78—C79	111.4 (4)
C74—N3—Ag2	116.07 (16)	C78—C79—H79A	109.5
C74—N3—P3	121.52 (17)	C78—C79—H79B	109.5
C1—N4—Ag2	126.45 (17)	C78—C79—H79C	109.5
C4—N4—Ag2	127.68 (17)	H79A—C79—H79B	109.5
P2—N5—Ag3	117.67 (11)	H79A—C79—H79C	109.5
C65—N5—Ag3	116.54 (16)	H79B—C79—H79C	109.5
C65—N5—P2	123.84 (18)	C78—C80—H80A	109.5
C29—N6—Ag3	128.88 (16)	C78—C80—H80B	109.5
C32—N6—Ag3	125.85 (17)	C78—C80—H80C	109.5
N4—C1—P1	116.64 (19)	H80A—C80—H80B	109.5
N4—C1—C2	111.2 (2)	H80A—C80—H80C	109.5
C2—C1—P1	131.60 (19)	H80B—C80—H80C	109.5
C1—C2—H2	127.0	C77—C81—H81	119.4
C1—C2—C3	106.0 (2)	C82—C81—C77	121.3 (3)
C3—C2—H2	127.0	C82—C81—H81	119.4
C2—C3—H3	126.9	C81—C82—H82	119.6
C4—C3—C2	106.3 (2)	C81—C82—C83	120.8 (2)
C4—C3—H3	126.9	C83—C82—H82	119.6
N4—C4—P2	119.99 (19)	C75—C83—N2	124.1 (2)
N4—C4—C3	110.9 (2)	C82—C83—N2	117.8 (2)
C3—C4—P2	128.8 (2)	C82—C83—C75	118.0 (2)
C6—C5—H5	120.3	C85—C84—H84	119.9
C6—C5—C10	119.4 (3)	C85—C84—C92	120.3 (3)
C10—C5—H5	120.3	C92—C84—H84	119.9
C5—C6—H6	119.9	C84—C85—H85	118.7
C7—C6—C5	120.3 (3)	C84—C85—C86	122.5 (3)
C7—C6—H6	119.9	C86—C85—H85	118.7
C6—C7—H7	119.7	C85—C86—C87	119.4 (2)
C6—C7—C8	120.5 (3)	C90—C86—C85	116.8 (2)
C8—C7—H7	119.7	C90—C86—C87	123.8 (3)
C7—C8—H8	120.1	C86—C87—H87	107.7
C7—C8—C9	119.8 (3)	C86—C87—C88	110.4 (2)
C9—C8—H8	120.1	C88—C87—H87	107.7
C8—C9—H9	120.1	C89—C87—C86	113.5 (2)
C8—C9—C10	119.9 (3)	C89—C87—H87	107.7
C10—C9—H9	120.1	C89—C87—C88	109.7 (2)
C5—C10—P2	121.3 (2)	C87—C88—H88A	109.5
C5—C10—C9	120.1 (3)	C87—C88—H88B	109.5
C9—C10—P2	118.3 (2)	C87—C88—H88C	109.5
C12—C11—H11	120.0	H88A—C88—H88B	109.5
C12—C11—C16	120.1 (3)	H88A—C88—H88C	109.5
C16—C11—H11	120.0	H88B—C88—H88C	109.5
C11—C12—H12	119.8	C87—C89—H89A	109.5
C13—C12—C11	120.5 (3)	C87—C89—H89B	109.5
C13—C12—H12	119.8	C87—C89—H89C	109.5
C12—C13—H13	119.9	H89A—C89—H89B	109.5
C12—C13—C14	120.2 (3)	H89A—C89—H89C	109.5
C14—C13—H13	119.9	H89B—C89—H89C	109.5
C13—C14—H14	120.1	C86—C90—H90	119.0
C15—C14—C13	119.7 (3)	C86—C90—C91	122.0 (3)
C15—C14—H14	120.1	C91—C90—H90	119.0
C14—C15—H15	119.9	C90—C91—H91	119.6
C14—C15—C16	120.3 (3)	C90—C91—C92	120.8 (3)
C16—C15—H15	119.9	C92—C91—H91	119.6
C11—C16—P2	119.9 (2)	C84—C92—N1	124.2 (2)
C11—C16—C15	119.2 (2)	C91—C92—N1	118.1 (2)
C15—C16—P2	120.8 (2)	C91—C92—C84	117.6 (2)
C18—C17—H17	120.1	O1—S1—C93	102.49 (17)
C18—C17—C22	119.7 (3)	O2—S1—O1	115.07 (17)
C22—C17—H17	120.1	O2—S1—C93	103.74 (15)
C17—C18—H18	119.9	O3—S1—O1	114.51 (16)
C19—C18—C17	120.2 (3)	O3—S1—O2	115.63 (16)
C19—C18—H18	119.9	O3—S1—C93	102.85 (16)
C18—C19—H19	119.8	F1—C93—S1	111.9 (2)
C18—C19—C20	120.4 (3)	F1—C93—F2	107.0 (3)
C20—C19—H19	119.8	F2—C93—S1	111.3 (2)
C19—C20—H20	120.1	F3—C93—S1	111.7 (3)
C19—C20—C21	119.9 (3)	F3—C93—F1	107.5 (3)
C21—C20—H20	120.1	F3—C93—F2	107.2 (3)
C20—C21—H21	120.1	C2A—C1A—H	119.7
C22—C21—C20	119.8 (3)	C2A—C1A—C6A	120.6 (14)
C22—C21—H21	120.1	C6A—C1A—H	119.7
C17—C22—P1	118.0 (2)	C1A—C2A—HA	119.6
C21—C22—P1	122.1 (2)	C3A—C2A—C1A	120.8 (12)
C21—C22—C17	119.9 (2)	C3A—C2A—HA	119.6
C24—C23—H23	120.0	C2A—C3A—HB	120.5
C28—C23—H23	120.0	C2A—C3A—C4A	119.0 (11)
C28—C23—C24	119.9 (3)	C4A—C3A—HB	120.5
C23—C24—H24	119.8	C3A—C4A—HC	119.4
C25—C24—C23	120.4 (3)	C3A—C4A—C5A	121.2 (13)
C25—C24—H24	119.8	C5A—C4A—HC	119.4
C24—C25—H25	120.0	C4A—C5A—HD	120.5
C26—C25—C24	120.0 (3)	C6A—C5A—C4A	119.1 (14)
C26—C25—H25	120.0	C6A—C5A—HD	120.5
C25—C26—H26	119.9	C1A—C6A—HE	120.4
C25—C26—C27	120.2 (3)	C5A—C6A—C1A	119.3 (14)
C27—C26—H26	119.9	C5A—C6A—HE	120.4
C26—C27—H27	120.0	C2B—C1B—HF	120.2
C26—C27—C28	120.0 (3)	C2B—C1B—C6B	119.6 (11)
C28—C27—H27	120.0	C6B—C1B—HF	120.2
C23—C28—P1	121.2 (2)	C1B—C2B—HG	119.7
C23—C28—C27	119.4 (2)	C3B—C2B—C1B	120.7 (11)
C27—C28—P1	118.9 (2)	C3B—C2B—HG	119.7
N6—C29—P3	121.19 (19)	C2B—C3B—HH	120.2
N6—C29—C30	110.9 (2)	C2B—C3B—C4B	119.6 (11)
C30—C29—P3	127.4 (2)	C4B—C3B—HH	120.2
C29—C30—H30	126.6	C3B—C4B—HI	119.5
C29—C30—C31	106.7 (2)	C3B—C4B—C5B	120.9 (10)
C31—C30—H30	126.6	C5B—C4B—HI	119.5
C30—C31—H31	127.2	C4B—C5B—HJ	120.7
C32—C31—C30	105.6 (2)	C6B—C5B—C4B	118.6 (10)
C32—C31—H31	127.2	C6B—C5B—HJ	120.7
N6—C32—P4	118.35 (18)	C1B—C6B—HK	119.7
N6—C32—C31	111.5 (2)	C5B—C6B—C1B	120.6 (11)
C31—C32—P4	129.86 (19)	C5B—C6B—HK	119.7
C34—C33—H33	120.2	C2C—C1C—HL	120.0
C34—C33—C38	119.5 (3)	C6C—C1C—HL	120.0
C38—C33—H33	120.2	C6C—C1C—C2C	120.0 (10)
C33—C34—H34	120.2	C1C—C2C—HM	120.5
C35—C34—C33	119.6 (3)	C3C—C2C—C1C	119.0 (11)
C35—C34—H34	120.2	C3C—C2C—HM	120.5
C34—C35—H35	119.3	C2C—C3C—HN	119.8
C34—C35—C36	121.5 (3)	C2C—C3C—C4C	120.3 (13)
C36—C35—H35	119.3	C4C—C3C—HN	119.8
C35—C36—H36	120.4	C3C—C4C—HO	120.0
C35—C36—C37	119.2 (3)	C3C—C4C—C5C	120.1 (14)
C37—C36—H36	120.4	C5C—C4C—HO	120.0
C36—C37—H37	120.1	C4C—C5C—HP	120.3
C36—C37—C38	119.8 (3)	C6C—C5C—C4C	119.4 (11)
C38—C37—H37	120.1	C6C—C5C—HP	120.3
C33—C38—P4	117.5 (2)	C1C—C6C—HQ	119.5
C37—C38—P4	122.1 (2)	C5C—C6C—C1C	121.1 (10)
C37—C38—C33	120.3 (2)	C5C—C6C—HQ	119.5
C40—C39—H39	120.1	C2D—C1D—HR	119.5
C44—C39—H39	120.1	C6D—C1D—HR	119.5
C44—C39—C40	119.7 (3)	C6D—C1D—C2D	121.0 (9)
C39—C40—H40	119.8	C1D—C2D—HS	119.9
C41—C40—C39	120.4 (3)	C3D—C2D—C1D	120.2 (10)
C41—C40—H40	119.8	C3D—C2D—HS	119.9
C40—C41—H41	119.9	C2D—C3D—HT	120.5
C42—C41—C40	120.2 (3)	C2D—C3D—C4D	118.9 (11)
C42—C41—H41	119.9	C4D—C3D—HT	120.5
C41—C42—H42	119.9	C3D—C4D—HU	119.8
C41—C42—C43	120.2 (3)	C3D—C4D—C5D	120.4 (11)
C43—C42—H42	119.9	C5D—C4D—HU	119.8
C42—C43—H43	120.0	C4D—C5D—HV	120.1
C42—C43—C44	120.0 (3)	C6D—C5D—C4D	119.8 (10)
C44—C43—H43	120.0	C6D—C5D—HV	120.1
C39—C44—P4	122.8 (2)	C1D—C6D—C5D	119.7 (10)
C39—C44—C43	119.4 (3)	C1D—C6D—HW	120.2
C43—C44—P4	117.7 (2)	C5D—C6D—HW	120.2
C46—C45—H45	119.9	C2E—C1E—HX	118.8
C46—C45—C50	120.3 (3)	C6E—C1E—HX	118.8
C50—C45—H45	119.9	C6E—C1E—C2E	122.5 (7)
C45—C46—H46	120.2	C1E—C2E—HY	120.6
C47—C46—C45	119.6 (3)	C1E—C2E—C3E	118.9 (7)
C47—C46—H46	120.2	C3E—C2E—HY	120.6
C46—C47—H47	119.9	C2E—C3E—HZ	121.6
C48—C47—C46	120.2 (3)	C2E—C3E—C4E	116.9 (8)
C48—C47—H47	119.9	C4E—C3E—HZ	121.6
C47—C48—H48	119.7	C3E—C4E—H1	117.8
C47—C48—C49	120.5 (3)	C5E—C4E—C3E	124.4 (6)
C49—C48—H48	119.7	C5E—C4E—H1	117.8
C48—C49—H49	120.2	C4E—C5E—H4	122.1
C50—C49—C48	119.6 (3)	C4E—C5E—C6E	115.7 (8)
C50—C49—H49	120.2	C6E—C5E—H4	122.1
C45—C50—P3	117.0 (2)	C1E—C6E—C5E	121.6 (9)
C49—C50—P3	123.1 (2)	C1E—C6E—H10	119.2
C49—C50—C45	119.8 (2)	C5E—C6E—H10	119.2
C52—C51—H51	119.9	C2F—C1F—H16	117.7
C52—C51—C56	120.3 (3)	C2F—C1F—C6F	125 (2)
C56—C51—H51	119.9	C6F—C1F—H16	117.7
C51—C52—H52	120.1	C1F—C2F—H22	120.2
C53—C52—C51	119.8 (3)	C1F—C2F—C3F	120 (2)
C53—C52—H52	120.1	C3F—C2F—H22	120.2
C52—C53—H53	120.1	C2F—C3F—H28	122.9
C54—C53—C52	119.9 (3)	C4F—C3F—C2F	114 (2)
C54—C53—H53	120.1	C4F—C3F—H28	122.9
C53—C54—H54	119.6	C3F—C4F—H29	116.2
C53—C54—C55	120.9 (3)	C3F—C4F—C5F	128 (2)
C55—C54—H54	119.6	C5F—C4F—H29	116.2
C54—C55—H55	120.3	C4F—C5F—H32	122.8
C56—C55—C54	119.4 (3)	C4F—C5F—C6F	114.5 (19)
C56—C55—H55	120.3	C6F—C5F—H32	122.8
C51—C56—P3	119.9 (2)	C1F—C6F—C5F	118.7 (19)
C55—C56—P3	119.9 (2)	C1F—C6F—H38	120.6
C55—C56—C51	119.7 (2)	C5F—C6F—H38	120.6
C58—C57—H57	119.4	C2G—C1G—H44	119.9
C65—C57—H57	119.4	C6G—C1G—H44	119.9
C65—C57—C58	121.3 (3)	C6G—C1G—C2G	120.3 (4)
C57—C58—H58	119.3	C1G—C2G—H50	119.9
C59—C58—C57	121.4 (3)	C3G—C2G—C1G	120.3 (4)
C59—C58—H58	119.3	C3G—C2G—H50	119.9
C58—C59—C60	121.8 (3)	C2G—C3G—H56	120.0
C58—C59—C63	117.4 (3)	C4G—C3G—C2G	120.0 (4)
C63—C59—C60	120.7 (3)	C4G—C3G—H56	120.0
C59—C60—H60	107.3	C3G—C4G—H59	120.2
C59—C60—C62	110.2 (3)	C3G—C4G—C5G	119.6 (4)
C61—C60—C59	113.2 (3)	C5G—C4G—H59	120.2
C61—C60—H60	107.3	C4G—C5G—H61	119.9
C61—C60—C62	111.3 (3)	C6G—C5G—C4G	120.3 (4)
C62—C60—H60	107.3	C6G—C5G—H61	119.9
C60—C61—H61A	109.5	C1G—C6G—C5G	119.6 (4)
C60—C61—H61B	109.5	C1G—C6G—H62	120.2
C60—C61—H61C	109.5	C5G—C6G—H62	120.2
H61A—C61—H61B	109.5	C2H—C1H—H65	120.3
H61A—C61—H61C	109.5	C6H—C1H—H65	120.3
H61B—C61—H61C	109.5	C6H—C1H—C2H	119.5 (3)
C60—C62—H62A	109.5	C1H—C2H—H68	120.0
C60—C62—H62B	109.5	C3H—C2H—C1H	119.9 (3)
C60—C62—H62C	109.5	C3H—C2H—H68	120.0
H62A—C62—H62B	109.5	C2H—C3H—H70	120.0
H62A—C62—H62C	109.5	C2H—C3H—C4H	120.0 (4)
H62B—C62—H62C	109.5	C4H—C3H—H70	120.0
C59—C63—H63	119.1	C3H—C4H—H71	120.0
C59—C63—C64	121.8 (3)	C5H—C4H—C3H	120.0 (3)
C64—C63—H63	119.1	C5H—C4H—H71	120.0
C63—C64—H64	119.8	C4H—C5H—H74	120.0
C63—C64—C65	120.4 (3)	C4H—C5H—C6H	120.0 (3)
C65—C64—H64	119.8	C6H—C5H—H74	120.0
C57—C65—N5	117.8 (2)	C1H—C6H—C5H	120.6 (3)
C57—C65—C64	117.6 (2)	C1H—C6H—H77	119.7
C64—C65—N5	124.6 (2)	C5H—C6H—H77	119.7
			
Ag1—N1—C92—C84	−156.8 (2)	C38—P4—C44—C43	−83.6 (2)
Ag1—N1—C92—C91	22.7 (3)	C38—C33—C34—C35	0.4 (4)
Ag1—N2—C83—C75	−133.5 (2)	C39—C40—C41—C42	1.8 (5)
Ag1—N2—C83—C82	42.8 (3)	C40—C39—C44—P4	178.2 (2)
Ag2—N3—C74—C66	26.8 (3)	C40—C39—C44—C43	0.0 (4)
Ag2—N3—C74—C73	−153.0 (2)	C40—C41—C42—C43	−1.5 (5)
Ag2—N4—C1—P1	11.4 (3)	C41—C42—C43—C44	0.4 (5)
Ag2—N4—C1—C2	−176.13 (17)	C42—C43—C44—P4	−177.9 (2)
Ag2—N4—C4—P2	−10.7 (3)	C42—C43—C44—C39	0.4 (4)
Ag2—N4—C4—C3	175.71 (17)	C44—P4—N1—Ag1	−127.40 (12)
Ag3—N5—C65—C57	27.7 (3)	C44—P4—N1—C92	68.7 (2)
Ag3—N5—C65—C64	−152.6 (2)	C44—P4—C32—N6	63.0 (2)
Ag3—N6—C29—P3	−5.6 (3)	C44—P4—C32—C31	−109.7 (2)
Ag3—N6—C29—C30	−178.02 (17)	C44—P4—C38—C33	−157.5 (2)
Ag3—N6—C32—P4	4.2 (3)	C44—P4—C38—C37	25.7 (3)
Ag3—N6—C32—C31	178.22 (16)	C44—C39—C40—C41	−1.1 (5)
P1—N2—C83—C75	22.8 (4)	C45—C46—C47—C48	1.8 (5)
P1—N2—C83—C82	−161.0 (2)	C46—C45—C50—P3	174.0 (2)
P1—C1—C2—C3	172.2 (2)	C46—C45—C50—C49	−1.5 (4)
P2—N5—C65—C57	−168.6 (2)	C46—C47—C48—C49	−1.3 (5)
P2—N5—C65—C64	11.2 (4)	C47—C48—C49—C50	−0.5 (5)
P3—N3—C74—C66	−160.1 (2)	C48—C49—C50—P3	−173.3 (2)
P3—N3—C74—C73	20.0 (4)	C48—C49—C50—C45	1.9 (4)
P3—C29—C30—C31	−173.11 (19)	C50—P3—N3—Ag2	−142.28 (13)
P4—N1—C92—C84	6.0 (4)	C50—P3—N3—C74	45.0 (2)
P4—N1—C92—C91	−174.4 (2)	C50—P3—C29—N6	−174.3 (2)
N1—P4—C32—N6	−60.1 (2)	C50—P3—C29—C30	−3.2 (3)
N1—P4—C32—C31	127.1 (2)	C50—P3—C56—C51	65.8 (2)
N1—P4—C38—C33	−25.6 (3)	C50—P3—C56—C55	−121.5 (2)
N1—P4—C38—C37	157.6 (2)	C50—C45—C46—C47	−0.4 (5)
N1—P4—C44—C39	−32.9 (3)	C51—C52—C53—C54	0.5 (5)
N1—P4—C44—C43	145.3 (2)	C52—C51—C56—P3	175.1 (2)
N2—P1—C1—N4	−52.6 (2)	C52—C51—C56—C55	2.4 (4)
N2—P1—C1—C2	136.8 (3)	C52—C53—C54—C55	0.9 (5)
N2—P1—C22—C17	−22.9 (3)	C53—C54—C55—C56	−0.7 (5)
N2—P1—C22—C21	155.1 (2)	C54—C55—C56—P3	−173.7 (3)
N2—P1—C28—C23	−25.3 (3)	C54—C55—C56—C51	−1.0 (5)
N2—P1—C28—C27	147.5 (2)	C56—P3—N3—Ag2	90.35 (15)
N3—P3—C29—N6	64.9 (2)	C56—P3—N3—C74	−82.3 (2)
N3—P3—C29—C30	−124.0 (2)	C56—P3—C29—N6	−59.5 (2)
N3—P3—C50—C45	46.2 (2)	C56—P3—C29—C30	111.6 (2)
N3—P3—C50—C49	−138.6 (2)	C56—P3—C50—C45	176.2 (2)
N3—P3—C56—C51	−165.8 (2)	C56—P3—C50—C49	−8.5 (3)
N3—P3—C56—C55	6.9 (3)	C56—C51—C52—C53	−2.2 (5)
N4—C1—C2—C3	1.2 (3)	C57—C58—C59—C60	179.9 (3)
N5—P2—C4—N4	75.6 (2)	C57—C58—C59—C63	2.4 (5)
N5—P2—C4—C3	−112.1 (2)	C58—C57—C65—N5	177.3 (3)
N5—P2—C10—C5	−140.8 (2)	C58—C57—C65—C64	−2.5 (5)
N5—P2—C10—C9	45.8 (2)	C58—C59—C60—C61	44.2 (5)
N5—P2—C16—C11	21.2 (3)	C58—C59—C60—C62	−81.1 (4)
N5—P2—C16—C15	−156.8 (2)	C58—C59—C63—C64	−1.9 (5)
N6—C29—C30—C31	−1.3 (3)	C59—C63—C64—C65	−0.8 (5)
C1—P1—N2—Ag1	−25.66 (14)	C60—C59—C63—C64	−179.5 (3)
C1—P1—N2—C83	176.5 (2)	C63—C59—C60—C61	−138.4 (4)
C1—P1—C22—C17	93.9 (2)	C63—C59—C60—C62	96.3 (4)
C1—P1—C22—C21	−88.2 (2)	C63—C64—C65—N5	−176.8 (3)
C1—P1—C28—C23	−139.2 (2)	C63—C64—C65—C57	3.0 (4)
C1—P1—C28—C27	33.6 (2)	C65—C57—C58—C59	−0.2 (5)
C1—N4—C4—P2	174.73 (18)	C66—C67—C68—C69	−176.9 (3)
C1—N4—C4—C3	1.1 (3)	C66—C67—C68—C72	1.1 (4)
C1—C2—C3—C4	−0.5 (3)	C67—C66—C74—N3	179.0 (3)
C2—C3—C4—P2	−173.3 (2)	C67—C66—C74—C73	−1.1 (4)
C2—C3—C4—N4	−0.4 (3)	C67—C68—C69—C70	−30.5 (5)
C4—P2—N5—Ag3	−38.90 (17)	C67—C68—C69—C71	96.3 (4)
C4—P2—N5—C65	157.6 (2)	C67—C68—C72—C73	−0.8 (4)
C4—P2—C10—C5	97.9 (2)	C68—C72—C73—C74	−0.4 (4)
C4—P2—C10—C9	−75.5 (2)	C69—C68—C72—C73	177.2 (3)
C4—P2—C16—C11	143.2 (2)	C72—C68—C69—C70	151.6 (3)
C4—P2—C16—C15	−34.8 (2)	C72—C68—C69—C71	−81.6 (4)
C4—N4—C1—P1	−173.92 (18)	C72—C73—C74—N3	−178.8 (3)
C4—N4—C1—C2	−1.5 (3)	C72—C73—C74—C66	1.3 (4)
C5—C6—C7—C8	−0.7 (5)	C74—C66—C67—C68	−0.1 (4)
C6—C5—C10—P2	−171.6 (2)	C75—C76—C77—C78	−176.2 (3)
C6—C5—C10—C9	1.8 (4)	C75—C76—C77—C81	0.8 (4)
C6—C7—C8—C9	0.7 (5)	C76—C75—C83—N2	174.0 (2)
C7—C8—C9—C10	0.5 (4)	C76—C75—C83—C82	−2.2 (4)
C8—C9—C10—P2	171.8 (2)	C76—C77—C78—C79	97.7 (4)
C8—C9—C10—C5	−1.8 (4)	C76—C77—C78—C80	−137.6 (3)
C10—P2—N5—Ag3	−155.35 (12)	C76—C77—C81—C82	−1.2 (4)
C10—P2—N5—C65	41.1 (3)	C77—C81—C82—C83	−0.1 (4)
C10—P2—C4—N4	−161.3 (2)	C78—C77—C81—C82	175.7 (3)
C10—P2—C4—C3	11.0 (3)	C81—C77—C78—C79	−79.1 (4)
C10—P2—C16—C11	−107.2 (2)	C81—C77—C78—C80	45.5 (4)
C10—P2—C16—C15	74.9 (2)	C81—C82—C83—N2	−174.7 (2)
C10—C5—C6—C7	−0.5 (5)	C81—C82—C83—C75	1.8 (4)
C11—C12—C13—C14	0.8 (4)	C83—C75—C76—C77	0.9 (4)
C12—C11—C16—P2	−179.2 (2)	C84—C85—C86—C87	−177.7 (2)
C12—C11—C16—C15	−1.2 (4)	C84—C85—C86—C90	1.5 (4)
C12—C13—C14—C15	0.1 (4)	C85—C84—C92—N1	178.6 (2)
C13—C14—C15—C16	−1.6 (4)	C85—C84—C92—C91	−0.9 (4)
C14—C15—C16—P2	−179.9 (2)	C85—C86—C87—C88	63.3 (3)
C14—C15—C16—C11	2.1 (4)	C85—C86—C87—C89	−173.1 (3)
C16—P2—N5—Ag3	79.20 (15)	C85—C86—C90—C91	−1.0 (4)
C16—P2—N5—C65	−84.3 (2)	C86—C90—C91—C92	−0.5 (4)
C16—P2—C4—N4	−47.6 (2)	C87—C86—C90—C91	178.2 (3)
C16—P2—C4—C3	124.8 (2)	C90—C86—C87—C88	−115.8 (3)
C16—P2—C10—C5	−12.9 (3)	C90—C86—C87—C89	7.8 (4)
C16—P2—C10—C9	173.6 (2)	C90—C91—C92—N1	−178.1 (3)
C16—C11—C12—C13	−0.2 (4)	C90—C91—C92—C84	1.4 (4)
C17—C18—C19—C20	0.5 (5)	C92—C84—C85—C86	−0.5 (4)
C18—C17—C22—P1	176.5 (2)	O1—S1—C93—F1	58.8 (3)
C18—C17—C22—C21	−1.4 (4)	O1—S1—C93—F2	−60.8 (3)
C18—C19—C20—C21	−1.2 (5)	O1—S1—C93—F3	179.3 (3)
C19—C20—C21—C22	0.6 (5)	O2—S1—C93—F1	−61.3 (3)
C20—C21—C22—P1	−177.1 (2)	O2—S1—C93—F2	179.1 (3)
C20—C21—C22—C17	0.8 (4)	O2—S1—C93—F3	59.3 (3)
C22—P1—N2—Ag1	93.13 (13)	O3—S1—C93—F1	177.9 (2)
C22—P1—N2—C83	−64.7 (2)	O3—S1—C93—F2	58.3 (3)
C22—P1—C1—N4	−175.42 (19)	O3—S1—C93—F3	−61.6 (3)
C22—P1—C1—C2	14.0 (3)	C1A—C2A—C3A—C4A	1 (3)
C22—P1—C28—C23	104.6 (2)	C2A—C1A—C6A—C5A	−1 (4)
C22—P1—C28—C27	−82.6 (2)	C2A—C3A—C4A—C5A	−4 (3)
C22—C17—C18—C19	0.8 (5)	C3A—C4A—C5A—C6A	4 (4)
C23—C24—C25—C26	1.4 (4)	C4A—C5A—C6A—C1A	−2 (4)
C24—C23—C28—P1	171.7 (2)	C6A—C1A—C2A—C3A	1 (4)
C24—C23—C28—C27	−1.1 (4)	C1B—C2B—C3B—C4B	−1 (2)
C24—C25—C26—C27	−1.8 (4)	C2B—C1B—C6B—C5B	0 (4)
C25—C26—C27—C28	0.7 (4)	C2B—C3B—C4B—C5B	1 (2)
C26—C27—C28—P1	−172.2 (2)	C3B—C4B—C5B—C6B	0 (3)
C26—C27—C28—C23	0.7 (4)	C4B—C5B—C6B—C1B	0 (3)
C28—P1—N2—Ag1	−139.46 (11)	C6B—C1B—C2B—C3B	1 (3)
C28—P1—N2—C83	62.7 (2)	C1C—C2C—C3C—C4C	−1 (4)
C28—P1—C1—N4	67.6 (2)	C2C—C1C—C6C—C5C	−3 (2)
C28—P1—C1—C2	−102.9 (3)	C2C—C3C—C4C—C5C	0 (5)
C28—P1—C22—C17	−152.5 (2)	C3C—C4C—C5C—C6C	0 (4)
C28—P1—C22—C21	25.5 (3)	C4C—C5C—C6C—C1C	2 (3)
C28—C23—C24—C25	0.1 (4)	C6C—C1C—C2C—C3C	2 (3)
C29—P3—N3—Ag2	−26.78 (17)	C1D—C2D—C3D—C4D	−2 (3)
C29—P3—N3—C74	160.54 (19)	C2D—C1D—C6D—C5D	−0.2 (18)
C29—P3—C50—C45	−74.3 (2)	C2D—C3D—C4D—C5D	0 (4)
C29—P3—C50—C49	101.0 (2)	C3D—C4D—C5D—C6D	1 (4)
C29—P3—C56—C51	−43.9 (2)	C4D—C5D—C6D—C1D	−1 (2)
C29—P3—C56—C55	128.8 (2)	C6D—C1D—C2D—C3D	2 (2)
C29—N6—C32—P4	−175.52 (17)	C1E—C2E—C3E—C4E	−0.2 (12)
C29—N6—C32—C31	−1.5 (3)	C2E—C1E—C6E—C5E	2.4 (19)
C29—C30—C31—C32	0.3 (3)	C2E—C3E—C4E—C5E	1.7 (10)
C30—C31—C32—P4	173.9 (2)	C3E—C4E—C5E—C6E	−1.2 (11)
C30—C31—C32—N6	0.7 (3)	C4E—C5E—C6E—C1E	−0.9 (13)
C32—P4—N1—Ag1	−12.15 (14)	C6E—C1E—C2E—C3E	−1.8 (18)
C32—P4—N1—C92	−176.1 (2)	C1F—C2F—C3F—C4F	−10 (6)
C32—P4—C38—C33	90.8 (2)	C2F—C1F—C6F—C5F	2 (8)
C32—P4—C38—C37	−86.0 (2)	C2F—C3F—C4F—C5F	7 (6)
C32—P4—C44—C39	−148.9 (2)	C3F—C4F—C5F—C6F	1 (6)
C32—P4—C44—C43	29.3 (2)	C4F—C5F—C6F—C1F	−5 (5)
C32—N6—C29—P3	174.11 (17)	C6F—C1F—C2F—C3F	6 (9)
C32—N6—C29—C30	1.7 (3)	C1G—C2G—C3G—C4G	0.6 (6)
C33—C34—C35—C36	−1.4 (5)	C2G—C1G—C6G—C5G	0.0 (7)
C34—C33—C38—P4	−176.0 (2)	C2G—C3G—C4G—C5G	−0.5 (7)
C34—C33—C38—C37	0.9 (4)	C3G—C4G—C5G—C6G	0.1 (7)
C34—C35—C36—C37	1.1 (5)	C4G—C5G—C6G—C1G	0.1 (7)
C35—C36—C37—C38	0.3 (5)	C6G—C1G—C2G—C3G	−0.3 (6)
C36—C37—C38—P4	175.5 (2)	C1H—C2H—C3H—C4H	−0.6 (6)
C36—C37—C38—C33	−1.2 (4)	C2H—C1H—C6H—C5H	0.0 (6)
C38—P4—N1—Ag1	104.55 (13)	C2H—C3H—C4H—C5H	0.6 (6)
C38—P4—N1—C92	−59.4 (2)	C3H—C4H—C5H—C6H	−0.3 (6)
C38—P4—C32—N6	177.48 (19)	C4H—C5H—C6H—C1H	0.0 (5)
C38—P4—C32—C31	4.7 (3)	C6H—C1H—C2H—C3H	0.3 (6)
C38—P4—C44—C39	98.2 (3)		

### Selected bond distances and angles for (I)

**Table:**

Atoms	Distance (Å)	Atoms	Angle (°)
Ag1–Ag2	3.2177 (2)	Ag1–Ag2–Ag3	65.677 (6)
Ag2–Ag3	2.8753 (3)	Ag2–Ag1–Ag3	52.186 (5)
Ag1–Ag3	3.3165 (2)	Ag2–Ag3–Ag1	62.138 (6)
Ag1–N1	2.168 (2)	N1–Ag1–N2	173.61 (8)
Ag1–N2	2.173 (2)	N3–Ag2–N4	170.50 (8)
Ag2–N3	2.127 (2)	N5–Ag3–N6	174.27 (8)
Ag2–N4	2.129 (2)	C1–N4–C4	105.6 (2)
Ag3–N5	2.136 (2)	C29–N6–C32	105.3 (2)
Ag3–N6	2.130 (2)	C1–P1–N2	104.88 (12)
P1–N2	1.610 (2)	C4–P2–N5	111.60 (11)
P2–N5	1.610 (2)	C29–P3–N3	111.65 (11)
P3–N3	1.608 (2)	C32–P4–N1	105.67 (11)
P4–N1	1.610 (3)	N1–Ag1–Ag2	121.87 (6)
N1–C92	1.413 (3)	N1–Ag1–Ag3	69.92 (6)
N2–C83	1.416 (3)	N2–Ag1–Ag2	64.04 (6)
N3–C74	1.425 (3)	N2–Ag1–Ag3	116.14 (6)
N5–C65	1.421 (3)	N3–Ag2–Ag1	120.57 (6)
		N3–Ag2–Ag3	92.63 (6)
		N5–Ag3–Ag1	62.138 (6)
		N5–Ag3–Ag2	95.83 (6)
		N4–Ag2–Ag1	65.22 (6)
		N4–Ag2–Ag3	83.00 (6)
		N6–Ag3–Ag1	62.87 (5)
		N6–Ag3–Ag2	81.53 (6)

### Selected bond distances and angles

**Table:**

Atoms	Distance (Å)	Atoms	Angle (°)
Ag1–Ag2	3.2175 (4)	Ag1–Ag2–Ag3	65.679 (8)
Ag2–Ag3	2.8753 (2)	Ag2–Ag1–Ag3	52.187 (8)
Ag1–Ag3	3.3165 (4)	Ag2–Ag3–Ag1	62.134 (8)
Ag1–N1	2.167 (3)	N1–Ag1–N2	173.69 (12)
Ag1–N2	2.174 (3)	N3–Ag2–N4	170.48 (12)
Ag2–N3	2.128 (3)	N5–Ag3–N6	174.34 (12)
Ag2–N4	2.132 (3)	C1–N4–C4	106.0 (3)
Ag3–N5	2.140 (3)	C29–N6–C32	105.5 (3)
Ag3–N6	2.135 (3)	P1–N2–C84	125.4 (3)
P1–N2	1.608 (3)	P2–N5–C66	123.9 (3)
P2–N5	1.603 (3)	P4–N1–C33	124.8 (3)
P3–N3	1.605 (3)	P3–N3–C75	121.4 (3)
P4–N1	1.610 (3)	N1–Ag1–Ag2	121.84 (3)
N1–C93	1.416 (5)	N1–Ag1–Ag3	69.89 (8)
N2–C84	1.418 (5)	N2–Ag1–Ag2	63.96 (8)
N3–C75	1.424 (5)	N2–Ag1–Ag3	63.96 (8)
N5–C66	1.420 (5)	N3–Ag2–Ag1	116.07 (8)
		N3–Ag2–Ag3	120.29 (8)
		N5–Ag3–Ag1	92.53 (8)
		N5–Ag3–Ag2	121.32 (9)
		N4–Ag2–Ag1	65.30 (8)
		N4–Ag2–Ag3	83.09 (8)
		N6–Ag3–Ag1	62.00 (8)
		N6–Ag3–Ag2	81.74 (8)

### Summary of short contacts

**Table:**

Atoms	Length
H36-C76	2.728
H36-C84	2.586
H53-O3	2.549
H14-O1	2.601
H81A-H6C	2.28
